# Temporal Dissection of Rate Limiting Transcriptional Events Using Pol II ChIP and RNA Analysis of Adrenergic Stress Gene Activation

**DOI:** 10.1371/journal.pone.0134442

**Published:** 2015-08-05

**Authors:** Daniel P. Morris, Beilei Lei, Lawrence D. Longo, Karol Bomsztyk, Debra A. Schwinn, Gregory A. Michelotti

**Affiliations:** 1 Center for Perinatal Biology, Loma Linda University, Loma Linda, California, United States of America; 2 Department of Anesthesiology, Duke University Medical Center, Durham, North Carolina, United States of America; 3 Department of Medicine, University of Washington, Seattle, Washington, United States of America; 4 Department of Anesthesiology, University of Iowa Carver College of Medicine, Iowa City, Iowa, United States of America; 5 Department of Pharmacology, University of Iowa Carver College of Medicine, Iowa City, Iowa, United States of America; 6 Department of Biochemistry, University of Iowa Carver College of Medicine, Iowa City, Iowa, United States of America; 7 Department of Medicine, Division of Gastroenterology, Duke University Medical Center, Durham, North Carolina, United States of America; Harvard Medical School, UNITED STATES

## Abstract

In mammals, increasing evidence supports mechanisms of co-transcriptional gene regulation and the generality of genetic control subsequent to RNA polymerase II (Pol II) recruitment. In this report, we use Pol II Chromatin Immunoprecipitation to investigate relationships between the mechanistic events controlling immediate early gene (IEG) activation following stimulation of the α_1a_-Adrenergic Receptor expressed in rat-1 fibroblasts. We validate our Pol II ChIP assay by comparison to major transcriptional events assessable by microarray and PCR analysis of precursor and mature mRNA. Temporal analysis of Pol II density suggests that reduced proximal pausing often enhances gene expression and was essential for Nr4a3 expression. Nevertheless, for Nr4a3 and several other genes, proximal pausing delayed the time required for initiation of productive elongation, consistent with a role in ensuring transcriptional fidelity. Arrival of Pol II at the 3’ cleavage site usually correlated with increased polyadenylated mRNA; however, for Nfil3 and probably Gprc5a expression was delayed and accompanied by apparent pre-mRNA degradation. Intragenic pausing not associated with polyadenylation was also found to regulate and delay Gprc5a expression. Temporal analysis of Nr4a3, Dusp5 and Nfil3 shows that transcription of native IEG genes can proceed at velocities of 3.5 to 4 kilobases/min immediately after activation. Of note, all of the genes studied here also used increased Pol II recruitment as an important regulator of expression. Nevertheless, the generality of co-transcriptional regulation during IEG activation suggests temporal and integrated analysis will often be necessary to distinguish causative from potential rate limiting mechanisms.

## Introduction

During this past decade, the transcription factor-centric view of gene regulation has increasingly been recognized as inadequate [1] and has given way to a more expansive view that acknowledges genetic control associated with virtually every step of transcription [2], as well as selective control of translation [3]. Central to current understanding of the transcriptional process is the role of the carboxy-terminal domain (CTD) of RNA polymerase II in integrating transcriptional signals using regulatory factors from promoter-associated mediator to the 3’ end processing machinery [4–8]. Early studies focused on the role of CTD phosphorylation in recruiting mRNA processing machinery to the pre-mRNA and established that most processing was co-transcriptional [9]. More recent genome wide analysis has driven the expanding view of transcriptional regulatory complexity. Among the most transformative of these findings is the presence of a transcriptionally engaged polymerase that has paused in the promoter proximal region of many active and inactive genes [10–15]. The generality of promoter proximal pausing (PPP) directly challenges the paradigm that transcription factors induce gene activation through Pol II recruitment, and demonstrates that mechanisms subsequent to Pol II recruitment and initiation are important regulators of gene expression [1]. Indeed, regulation of proximal pausing is probably the function of some prototypic transcription factors including Myc, which appears to drive gene activation through abrogation of pausing [14]. Beyond proximal pausing and the established regulatory mechanisms of alternative splicing, alternative 3’ end formation and mRNA stability, recent studies suggest additional post-initiation mechanisms including degradation of pre-mRNA during both elongation [16, 17] and 3’ end formation [18, 19], as well as poorly defined functions associated with DNA looping [4, 20, 21], antisense transcription [22] and non-coding RNAs [23].

Despite considerable study, the biological function of the conserved immediate early genes (IEGs) is poorly understood, at least in part due to complexity within the acute activation process that prevents clear association with specific signaling pathways. Consequently, concordant expression patterns often do not demonstrate commonality of mechanism. While functions related to acute IEG expression presumably explain why these genes are usually short and activated within minutes [24–26], the biological significance of rapid expression has been difficult to establish. Even in the tractable yeast model, *S*. *Cerevisiae*, the function of the acute environmental stress response genes is uncertain, as inactivation of individual or many of these genes has little impact on the ability of this organism to survive lethal insult [27–29]. Importantly, evidence suggests the environmental stress response genes of yeast functions to precondition cells exposed to an initial stress prior to a second potentially lethal stress, even when the two stressors are different [29]. In mammals, a similar acute preconditioning response was discovered in 1986 by McMurry and coworkers, who demonstrated that a brief blockage of blood flow to the heart was strongly protective against the damage induced by a subsequent, longer insult [30]. The phenomenon of acute ischemic preconditioning has since been shown be a part of a general protective mechanism that can be activated by many insults [31] within the time frame of IEG activation.

One of the earliest and most established pharmacologic activators of cardiac preconditioning is phenylephrine (PE), a specific agonist of the α_1_-Adrenergic Receptors (α_1_ARs) [32, 33]. All three members of the α_1_AR family (α_1a_, α_1b_ and α_1d_ ARs) are Gq-coupled receptors that signal in part through activation of PLCβ [34]. This Gq-activated phospholipase cleaves phosphatidylinositol into the second messengers, diacylglycerol and inositol triphosphate, resulting in acute release of intracellular calcium stores and Protein Kinase C activation. However, a complex array of additional pathways are also activated by the α_1_ARs [35] including regulation of ion channels [36] and transactivation of endothelial growth factor receptor [37]. As part of the fight or flight response [38], α_1_AR stimulation evokes responses such as vessel contraction within fractions of a second [39], while other aspects of signaling unfold over minutes (e.g. mitogen activated protein kinases; MAPKs), hours (e.g. gene expression) or days (e.g. injury recovery). The α_1a_AR subtype in particular is linked to cardioprotection [40, 41] and while the biological basis is uncertain, the α_1a_AR activates a robust genetic response in both myocytes [42, 43] and expression models [44, 45]. Given this range of acute and chronic effects, it is obvious that the biological basis of α_1a_AR function involves temporally evolving interaction between the signaling and genetic cascades.

While methods are available to dissect temporal relationships between signaling factors (e.g. phospho-specific antibodies), temporal dissection of the complex mechanisms of gene regulation has been less assessable. To gain insight into the importance of specific mechanisms of gene regulation as well as the impact of each mechanism on the time required for IEG gene expression, we used Pol II Chromatin Immunoprecipitation (Pol II ChIP) to analyze the transcriptional response induced by stimulation of the α_1a_AR. Combined with analysis of changes in product RNA levels, Pol II ChIP allowed a temporal, integrated description of the rate-limiting mechanisms of induced gene expression. Our data supports a regulatory role for recently described co-transcriptional mechanisms including PPP [6, 10], internal transcriptional blocks [16, 17], and polyadenylation associated pre-mRNA degradation [18, 19] as well as demonstrating the initial transcriptional velocity on activated mammalian IEGs is very high and approaches the rate observed on long genes [46]. The apparent generality of co-transcriptional mechanisms suggests any particular genetic mechanism will only be rate limiting in specific situations [1] and justifies integrated temporal analysis, as this approach reveals the timing and relative importance of regulated genetic mechanisms. This depth of information is also necessary to identify upstream signaling cascade responsible for activation. Given conservation of IEG expression, these results should extend to in vivo stress responses of direct relevance to human health.

## Experimental Procedures

### Materials

Dulbecco’s Eagle Modified Medium and penicillin/streptomycin (15140–122) were from Gibco and heat inactivated fetal calf serum (SH30071.03) from Hyclone (Logan, Utah). Phenylephrine (P6126), PMSF (P7626), sodium orthovanadate (Na3VO4, S-6508), sodium deoxycholate (D-6750), glycogen (G-8751), bovine serum albumin (A-2153), formaldehyde (F1268), NaF, goat anti-mouse IgM (M-8644) were from Sigma-Aldrich (St. Louis, MO). Trizol and Platinum Taq from Invitrogen. Protein G agarose (1 719 416), Complete protease inhibitor tablets (1 836 145), Proteinase K (3 115 879), NP-40 (1 754 599) were from Roche, Indianapolis, IN. Herring sperm DNA (D181B) was from Promega (Madison, WI). H14 (MMS-134R) was from Biolegend (Dedham, MA) and H48 from Cell Signaling (Danvers, MA).

### Cell culture and treatment conditions

Isolation and characterization of clonal rat-1 fibroblasts stably expressing HA-tagged human α_1a_AR at 1.77±0.24 pmol/mg total protein has been described [47]. Cells were maintained under 5% CO_2_ at 37°C in growth medium containing Dulbecco’s Eagle Modified Medium supplemented with 10% fetal bovine serum, penicillin (100 U/ml), streptomycin (100 μg/ml) and 0.5 mg/ml G418 to maintain selection. Two days prior to use (42–50h), cells in mid-log phase were trypsinized and plated at 2 to 2.5 million cells per 15 cm dish in growth medium without G418. Mid-log phase cells at about 70% confluence and still in the growth medium under 5% CO_2_ at 37°C were stimulated by addition of filter sterilized phenylephrine (PE) stocks in water to a final concentration of 10^−5^ M, followed by gentle circular rotation to ensure mixing with minimal agitation. Cells were processed as indicated below.

### Isolation of total RNA

Media was poured from the plates and the remnant aspirated, prior to rapid addition of 10 ml of Trizol. Cells were then incubated with rotation for 20 to 40 minutes at room temperature prior to storage at -80°C. Remaining steps were performed as recommended by the manufacturer employing a tabletop centrifuge. Purified RNA resuspended to 1μg/μl in water and stored as subaliquots at -80°C.

### Agilent microarray analysis of polyadenylated RNA

RNA quality checks, cDNA preparation and Microarray analysis were performed at the DUKE IGSP Microarray Facility. Cy5 labeled sample cDNA and Cy3 labeled reference cDNA were made by reverse transcription with oligo-dT primers and were hybridized to a DNA chip printed with the Rat Operon 3.0 (RO27K) oligo set containing 26,962 probes. Reference cDNA was a composite of RNAs from multiple time points to ensure the presence of all upregulated mRNAs. The data were Lowess normalized and background-subtracted using Genespring (Agilent, Santa Clara, CA), normalized to the average of 3 untreated controls and deposited in NCBI's Gene Expression Omnibus at GEO Series accession number: http://www.ncbi.nlm.nih.gov/geo/query/acc.cgi?acc=GSE59955.

### High Efficiency Chromatin Immunoprecipitation

Chromatin Immunoprecipitation was performed as previously described [48] with minor modification. Briefly, media was poured and then aspirated from plates following stimulation with PE for the indicated times. The cells were fixed with 1% formaldehyde in PBS with gentle agitation for 10 minutes at 25°C after which the formaldehyde was removed and crosslinking stopped by incubation with 125 mM glycine in PBS for 5 min at 25°C. Following solution removal, plates were chilled on ice and the cells lysed by addition of 2 ml of cold lysis buffer [10 mM Tris, pH 7.5 at 25°C, 0.5% SDS plus protease inhibitors (1 mM PMSF and 5X complete protease inhibitor cocktail)and phosphatase inhibitors (1 mM Na3VO4, 10 mM Na4P2O7, and 10 mM NaF)]. The cells were rapidly scraped from the plate and the solution (~2.3 mL) was transferred to a short, 5 ml tube in ice water and sonication to an average fragment size of ~1000 to 1100 base pairs using twelve 5 sec bursts on a Misonex 3000 with a microtip probe (Farmingdale, NY). Precipitate formation was prevented through judicious warming regulated by varying ice water immersion. Following clarification by centrifugation at 10,000 x g for 5 min at 5°C, supernatants were diluted about 20-fold with 38 ml of cold IP dilution buffer (10 mM Tris, pH 7.5 at 25°C, 1% NP40, 0.5% sodium deoxycholate, 150 mM NaCl, protease and phosphatase inhibitors as above). The combined solution approximates ChIP buffer (IP dilution buffer with 0.025% SDS). Subaliquoted samples were frozen with liquid nitrogen and stored at -80°C until use. Each 1.6 ml of extract represents about 250,000 rat-1 cells.

For ChIP, protein-G agarose beads in IP dilution buffer were bound to goat anti-mouse IgM at 1 μg/ul beads and then pre-loaded with excess H14 at ~3μg/μl beads [48]. Both Preclearing and H14 beads were prepared and equilibrated with SDS blocking buffer [ChIP buffer with 1 mg/ml bovine serum albumin and 1 mg/ml herring-sperm DNA]. Extract was precleared for 1h with 15 μl protein-G beads/1.6 ml and then ChIPed for 2h with 10 μl H14 beads/1.6 ml, both at 4°C with inversion mixing. Following immunoprecipitation, beads were washed 4 times with 1.0 ml ChIP buffer per 10 μl protein-G beads, each time using 5 min inversions and recovery by centrifugation at 500xg for 1 min. Washed beads were suspended in 100 μl of ChIP buffer to which was added 2.5 μl 10% SDS and 5 μl of 10 mg/ml proteinase K. Samples were mixed well and incubated 2 hours at 55°C with occasional mixing and then overnight at 65°C. Sample beads were spun and the supernatant was collected followed by a second extraction with 100 μl TE (10 mM Tris, pH 8 and 1 mM EDTA) that was combined with first supernatant. Following phenol/Chloroform/IA and then chloroform extraction, 2 μl of 10 μg/μl glycogen was added and the precipitated with Na acetate/EtOH. Total genomic DNA was similarly prepared from ChIP extract without glycogen. ChIPs were resuspended in water, while genomic DNA was placed into TE and later diluted with water to appropriate concentrations.

### Analysis and quantitation of ChIP DNA by PCR

Primer sequences (S3 Table) were designed as described [48] using Oligo (Molecular Biology Insights, Cascade, CO) and obtained from Qiagen in unpurified salt free state. Primer locations are designated using the 5’ most bp (base pair) of the upstream primer. PCR was performed with Platinum Taq as recommended (Invitrogen) but with 500 nM primer concentrations using a PTC-225 Tetrad DNA Engine from M.J. Research (Waltham, MA). Taq was kept at -80°C for long term storage and kept at -20°C for no more than 1 month. Reactions were “hot” started by incubation for 1.5 min at 94°C, followed by 35 cycles of 0.5 min at 94°C, 0.5 min at 60°C and 1 min at 72°C plus a final 10 min extension at 72°C. Following agarose gel electrophoresis in an model A6 Owl gel apparatus (Thermo Scientific) with 25 well combs suited for use with a multichannel pipet, images of ethidium bromide stained PCR products were quantitated by densitometry with appropriate background subtraction. A two-fold dilution series of total genomic DNA, purified from ChIP extracts of untreated cells, was used to generate a non-linear response curve between input DNA and PCR product DNA. Profiles presented were chosen on the basis of representative behavior and, when appropriate, a quantitation curve that trended downward with decreasing input DNA. For each time point, the fraction of DNA precipitated by ChIP was estimated by interpolation between response curve values bracketing the sample signal. Accuracy was improved by averaging estimates from time points that displayed common behavior.

### Matrix ChIP analysis of Pol II density

The use of microplates to perform ChIP has been described in detail [49], and was adapted for use with ChIP extracts prepared as described above. For each time point 50 μl of extract (in triplicate) were added directly to 96 well plates coated with the anti-CTD 4H8 antibody and the matrix ChIP procedure applied as described [49]. To avoid SDS interference with SYBR Green qPCR, a 100-fold dilution of input ChIP extract was used as control and precipitation efficiency calculated relative to the combined average value from all time points. Matrix ChIP primers are given in S4 Table.

### TaqMan quantitative PCR

Total RNA prepared by Trizol extraction was treated with RNase-free DNase I (0.4 U per μl of RNA sample) for 30 minutes at 37°C, heat denatured at 65°C and purified over Sephadex G-25 columns. RNA concentrations and quality were confirmed by NanoDrop spectrophotometer ND-1000 (NanoDrop technologies; Wilmington, DE) and cDNA prepared using random primers in general or oligo-dT primers when stated. Quantitation of mRNAs was performed in a 48 well format on an ABI Step One Plus RT-PCR system using TaqMan appropriate primers (S5 Table) with each well containing cDNA from the equivalent of 30–300 ng of total RNA. Target gene quantitation was conducted according to the 2^–ΔΔCt^ method with expression normalized to the housekeeping gene RPL35. Basal values and periods of activated expression are included in S5 Table.

### Non-quantitative PCR analysis of total RNA

cDNA prepared using oligo-dT priming was analyzed with primers (S3 Table) and assay conditions used for Pol II ChIP analysis, using cDNA derived from the amount of total RNA in the indicated number of cells (12.5 ng of total RNA from 2,100 source cells).

### Statistical analysis

Prism software (GraphPad, La Jolla, CA) was used for statistical analysis. Error is given as standard deviation with the indicated exceptions where SEM was appropriate. Regression analysis was used to evaluate potential trends in Pol II density along the length of the gene. Regression analysis and one-way ANOVA with the Turkey post-test were used to establish difference between time points. Additional analysis used the Student t-test or two way ANOVA with post-test, as indicated.

## Results

### Identification of α_1a_AR regulated acute stress response genes

For all studies, an HA-α_1a_AR expressing rat-1 cell line was cultured in growth media and treated with 10^−5^ M PE to produce the characteristic antiproliferative and hypertrophic response of this receptor [37, 45, 50]. Microarray analysis of α_1a_AR-induced gene expression has been reported for rat-1 cells [45]; however, our analysis of transcriptional activation required detailed temporal information under the specific assay conditions chosen. Because this precludes replicated data, typical measures of statistical validation were inappropriate. Nevertheless, the time course provided a sensitive measure of gene activation, apparent on upregulated genes as a pattern of increasing mRNA followed by a the period of maximal expression (S1 Table). Remarkably, stimulation of the α_1a_AR activates most genes consistently upregulated by the severe stress of cardiac surgery [51–53] and by stimulation of native Gq-coupled endothelin receptors [54] in neonatal rat cardiomyocytes (S2 Table).

### Characteristics of the high efficiency Pol II ChIP assay and RNA analysis

In contrast to other measures of gene activation, Pol II ChIP reveals transcriptional responses as they occur by directly demonstrating increases in bound Pol II at desired points along a gene [55, 56]. Many studies have now shown that high efficiency Pol II ChIP directed at the S5 phosphorylated CTD effectively precipitates Pol II across transcribing genes at least through the polyadenylation site (see Discussion). Although highly sensitive, our Pol II ChIP procedure has limited resolution due to relatively large DNA fragments, which average about 1000 to 1100 base pairs and include fragments of 2000 base pairs. More generally, Pol II ChIP is inherently semi-quantitative and has not been validated by other technologies. Indeed, available evidence shows biological context can alter sonication-induced cleavage [57], implying changes in Pol II signal can reflect alternative cleavage as well as changes in Pol II density. For these reasons, the precision of Pol II ChIP is unverifiable and we have instead used a strategy of redundancy with multiple primer pairs to confirm polymerase density with reasonable accuracy using standard PCR and gel electrophoresis.

Because spatial and temporal separation of co-transcriptional regulatory events is easier on longer genes, our analysis has focused on long IEGs activated by the α1aAR stimulation (Table 1). Temporal analysis of changing RNA levels must be interpreted in relation to the environment of the target sequence and these target locations are indicated for both Agilent microarray and TaqMan qPCR analysis. It is important to note that microarray analysis was directed at polyadenylated messages through use of cDNA transcribed from Olig-dT primers. In contrast, TaqMan qPCR employed cDNA made using random primers (unless otherwise stated) and detected target sequence in both mature mRNAs and pre-mRNAs, generating an estimate of “total” RNA.

**Table 1:** IEGs Activated by Stimulation of α_1a_AR with 10^−5^ M PE and Analyzed in This Study.

Gene	Function	Gene ID	Length base pairs	Microarray^1^ * Target	TaqMan^2^ * Target
Fos	TF, oncogenic	314322	2867	exon 4T	exon 2, 3
Nr4a3	TF, pleiotropic	58853	39843	exon 8T	exon 4, 5
Nr4a1	TF, pleiotropic	79240	7784	exon 7T	exon 6, 7T
Nfil3	TF, pleiotropic	114519	~15000	exon 2T	exon 2T
Dusp5	phosphatase, MAPK	171109	13411	exon 4T	exon 2, 3
Gprc5a	GPCR, growth	312790	18911	exon 2	exon 2, 3

^1^Microarray used oligo-dT prime cDNA and measured poly-A mRNA.

^2^TaqMan qPCR used randomly primed cDNA unless otherwise stated.

*The designation “T” indicates a 3’ terminal exon.

### Analysis of the Fos activation using Pol II ChIP, microarrays and qPCR

**Fig 1:**
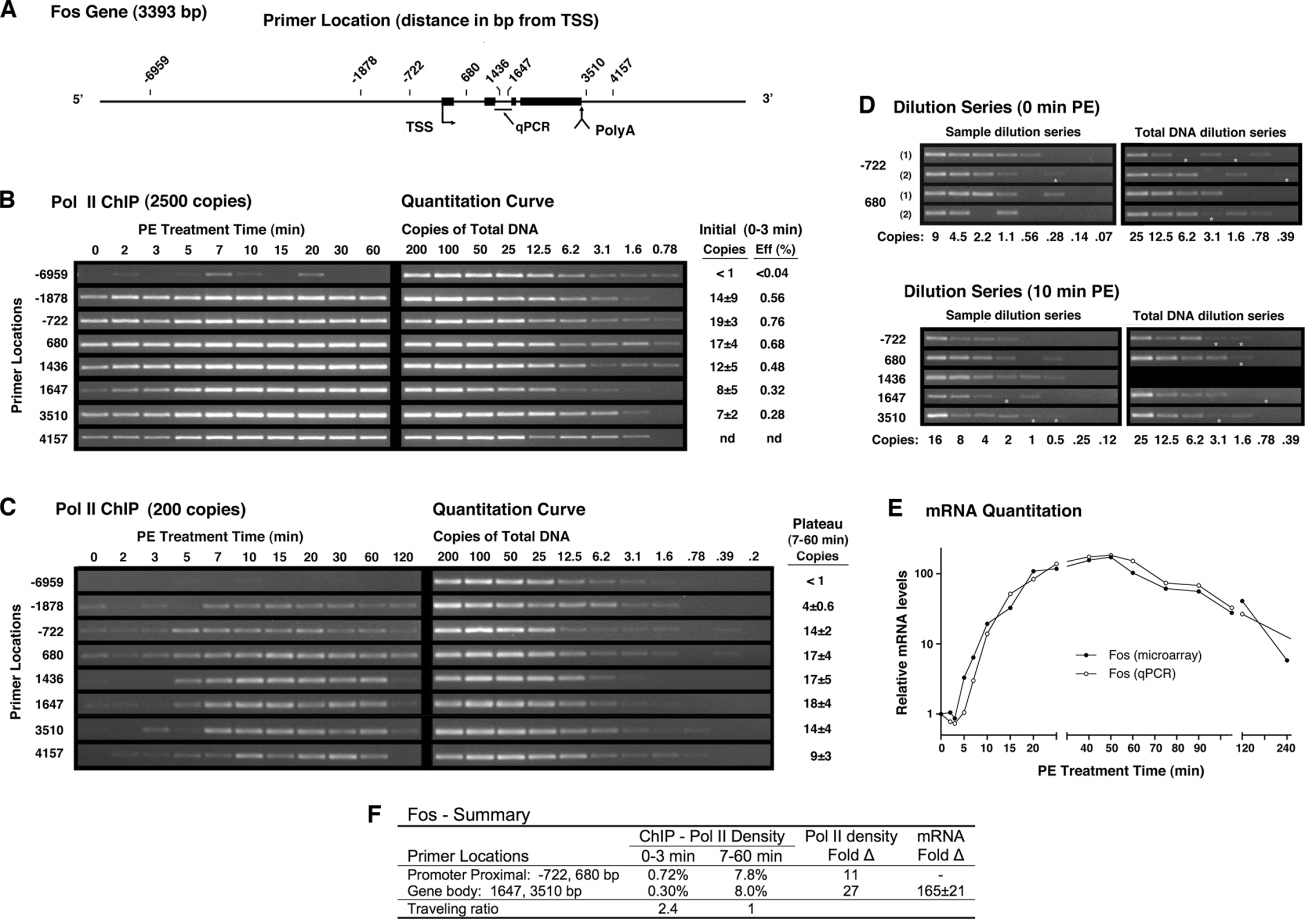
Temporal analysis of Fos activation using Pol II ChIP, Microarray and qPCR. Cells were stimulated with 10^−5^ M PE for the indicated times prior to preparation of ChIP extracts or total mRNA. (**A**) Schematic of the Fos gene showing sequence locations targeted by ChIP primer pairs (5’ end relative to annotated TSS) and the TaqMan qPCR primer set (bar). (**B**) Pol II density analyzed by ChIP with the H14 antibody as described in Methods. For each time point, the DNA ChIPed from extract equivalent to 1,250 cells (2500 genome copies) was analyzed by PCR using the indicated primers. PCR product was quantitated relative to dilution series with indicated genomic copies of DNA extracted from unstimulated cells (right). For each primer set, initial Pol II density (0–3 min.) is expressed as precipitated copies (mean±SD) and as percent precipitation efficiency relative to input genome copies (far right). (**C**) Pol II density during plateau of activated expression. ChIP DNA (200 genome copies) was analyzed as described above. For each primer set, average Pol II density during the plateau of activated expression (7–60 min.) is expressed as precipitated copies (mean±SD). (**D**) Dilution series of ChIPed DNA to zero copies is consistent with quantitative estimates of Pol II density. Sample and total image intensity were adjusted independently. Total genomic DNA is only to confirm single copy product formation. (*) Indicates low product is present. (**E**) Temporal analysis of relative Fos mRNA levels. Agilent microarray analysis (●) of polyadenylated mRNA (oligo-dT primed) and TaqMan qPCR analysis (○) of total mRNA (mRNA + pre-mRNA) using randomly primed cDNA. Both target locations listed in Table 1. (**F**) Summary of transcriptional activity. Pol II density expressed as percent precipitation efficiency relative to input copy number. TaqMan qPCR primer set was used to quantitate Fold-Δ in total mRNA relative to basal levels (S5 Table).

To demonstrate that our Pol II ChIP assay reproduces established co-transcriptional behavior, we analyzed the activation of Fos; a gene known to be regulated by abrogation of promoter proximal pausing [58, 59]. Using PCR primer pairs at positions summarized in the Fos gene schematic (Fig 1A), transcription was analyzed using DNA from 1/200 of a standard ChIP representing 1,250 cells (2,500 potentially precipitable copies of DNA) as input to each PCR reaction. These results (Fig 1B, left) are presented as a function of time following stimulation (horizontal axis) versus location along the gene (vertical axis) as indicated by the primer position. Visually, basal Pol II density appears higher in the promoter proximal region (primers at -722, 680 bp) than at more distal sites (1647, 3510 bp). Although, it may seem surprising that nearby primers (-1878, 1436 bp) reflect promoter proximal density, this is typical of our data for primers producing amplicons encompassed within 2,000 base pairs of PPP sites. Following receptor stimulation, rapid activation is apparent between 3 and 5 minutes as shown by the substantial increase in Pol II density at locations across the gene. Pol II density during the elevated plateau of activity from 7 to 60 minutes displayed saturating signal as shown by comparison to a dilution series of genomic input DNA from uninduced cells (Fig 1B, right). On the other hand, Pol II density prior to upregulation (0–3 min) can be estimated using these curves and is presented as both copy number and precipitation efficiency (Fig 1B, far right). As summarized in Fig 1F, upstream density at proximal primers was 0.72% (~18 copies) or about 2.4–fold higher than observed at distal primers where efficiency was 0.3% (~7.5 copies). Although for Fos this higher density almost certainly represents PPP, the ratio of proximal to distal Pol II density is often referred to as a traveling ratio in order to avoid mechanistic implication.

To quantitate transcription levels following Fos activation, Pol II density was reanalyzed using DNA from 1/2,500 of a standard Pol II ChIP (Fig 1C) representing material from 100 cells (200 potentially precipitable copies of DNA) as input to each PCR reaction. During the period of activated expression from 7 to 60 minutes, Pol II density was the same at promoter proximal and distal primers (Fig 1C) and displayed a combined ChIP precipitation efficiency of 8.0±1.0%. Indeed, within this activated plateau, analysis showed Pol II density to be statistically constant for all primers (Fig A in S1 Fig) and at each time point across the gene (Fig A in S2 Fig). Constant Pol II density following activation, demonstrates the traveling ratio on Fos was reduced from 2.4 to 1 (Fig 1F), consistent with abrogation of proximal pausing observed in other models [55, 56]. In addition, comparison of distal Pol II levels before and after gene activation revealed a 27-fold (8%/0.3%) increase in density that suggests a ~27-fold increase in transcription.

### Independent validation of Pol II ChIP quantitation

In general, ChIP primer sets were capable of producing amplicons from one copy of DNA, suggesting an independent method of validation based on serial dilution to zero copies; an approach currently employed in digital PCR. Following a Poisson distribution, PCR reactions receiving the indicated number of DNA copies (on average) have the subsequent probability of receiving no copies: 3.1 (4%), 1.6 (20%), 0.78 (45%), 0.39 (68%) and 0.2 (84%). This distribution is consistent with the genomic DNA dilution series from Fig 1B and 1C, where the input copies of DNA produced the indicated percentage of product absence: 3.1 (0%), 1.6 (0%), 0.78 (61%), 0.39 (75%) and 0.2 (100%). Applying this methodology to confirm the estimates of Pol II density obtained using quantitation curves, we observe that loss of PCR product occurs at dilutions of ChIPed DNA (Fig 1D) consistent with the quantitative estimates of copy number. Although reactions can fail and false positives can occur, dilution to zero, nevertheless, provides an estimate of copy number that is independent of PCR product intensity at higher genomic DNA concentrations.

### Comparison of Pol II and mRNA levels implies increased Fos mRNA stability

If Pol II ChIP experiments for Fos accurately reflect the initial wave of newly activated Pol II, then increasing mRNA should be evident shortly after the Pol II reaches the 3’end of the gene. Indeed, microarray analysis shows polyadenylated mRNA begins accumulating within 5 min of α_1a_AR stimulation (Fig 1E) concurrent with Pol II arrival (Fig 1B). Quantitative PCR, which in this case reports intron 2 excision (Fig 1A), shows an increase in spliced mRNA by 7 minutes. As random priming generates cDNA from both pre-mRNA and mature mRNA, relative expression estimated by qPCR represents the change in “total” mRNA containing the target sequence. Aside from this early temporal difference, Fos mRNA levels determined by microarray analysis and qPCR are similar (Fig 1E), including the maximal 165-fold increase reported by the qPCR measurement (Fig 1F). Because transcription (Pol II density) and mRNA levels (qPCR) display coordinated periods of stable activated expression, relative changes can be compared directly. For Fos, the observed transcriptional increase of 27-fold reported by Pol II ChIP is insufficient to account for the 165-fold upregulation of mRNA, suggesting increased stability contributed ~6-fold to the maximal increased mRNA levels (Fig 1F). As the increases in Fos transcription and mRNA have been redundantly established, this estimate provides a quantitative measure of the contribution of increased mRNA stability. Although the estimate depends on constant transcriptional velocity before and after stimulation, transcription in these experiments is probably rapid and fairly constant, as suggested by both our data (see below) and previously published results [46, 60, 61].

### Pol II ChIP analysis of Nr4a3

**Fig 2:**
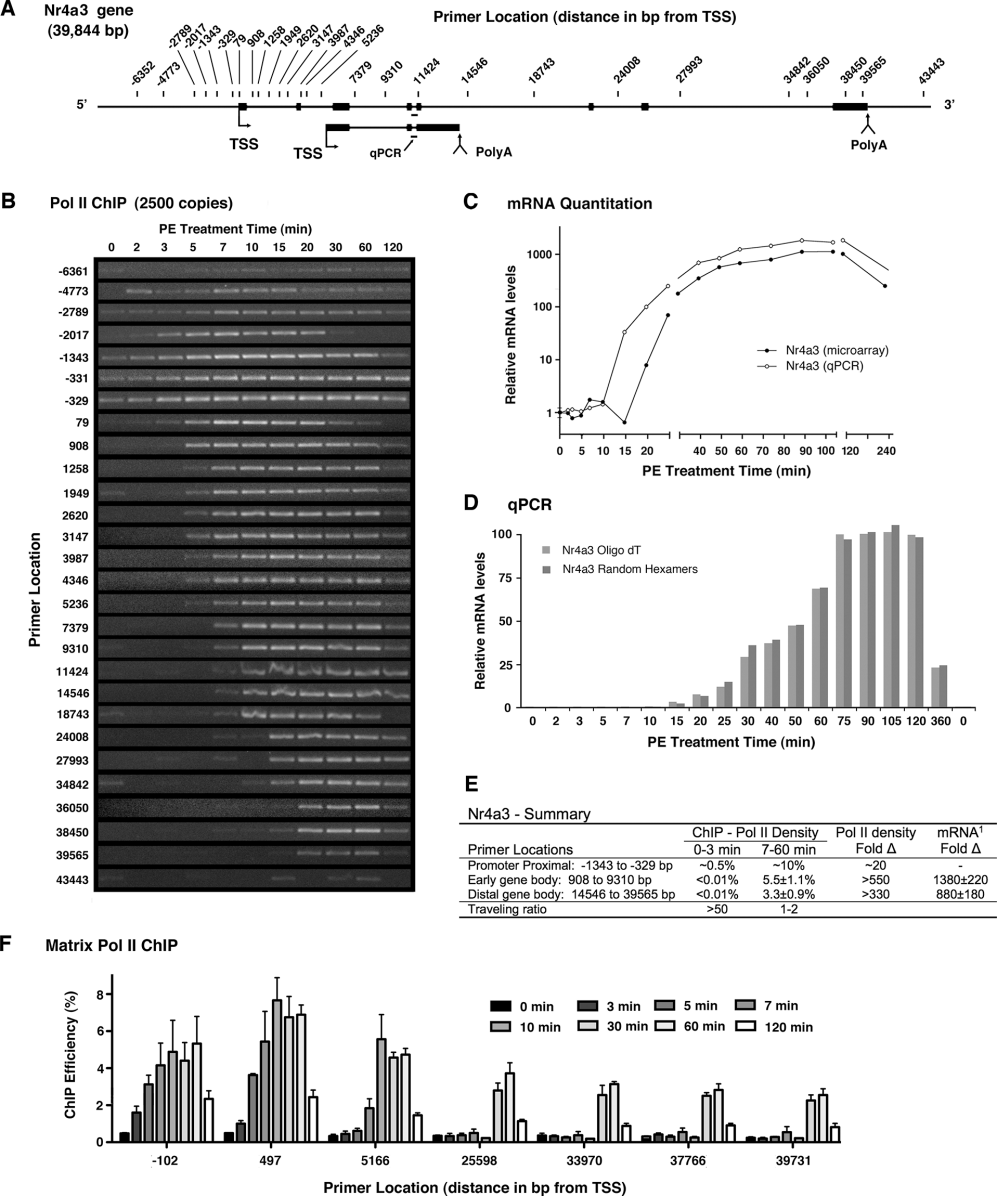
Analysis of Nr4a3 activation by Pol II ChIP, Matrix ChIP, Microarray and qPCR. Combined schematic (**A**) of long and short Nr4a3 isoforms showing locations of ChIP primers relative to the TSS of the longer form as well as the TaqMan qPCR primer set (bar). (**B**) Temporal Pol II ChIP analysis using DNA ChIPed from extract equivalent to 2500 genome copies. (**C**) Analysis of relative mRNA levels. Agilent microarray analysis (●) of polyadenylated mRNA and TaqMan qPCR analysis (○) of total mRNA (mRNA + pre-mRNA) using upstream primers spanning an intron common to long and short transcripts (see schematic). (**D**) Comparative TaqMan qPCR analysis with oligo dT and random hexamer primed cDNA. Analysis shows parallel formation of short polyadenylated mRNAs and total mRNA. Data are the averages of two experiment presented with a linear Y-axis to accent similarity. (**E**) Summary of transcriptional activity. Pol II density expressed as percent precipitation efficiency. ^1^mRNA measurements based on TaqMan qPCR for early total mRNA and microarray for polyadenylated long mRNA. (**F**) Matrix ChIP quantitation of Pol II density based on the 4H8 anti-CTD antibody and analyzed using qPCR (SYBR Green) at the indicated sites relative to annotated TSS (S4 Table).

One of the genes most upregulated by α_1a_AR stimulation was Nr4a3, a medically important [51, 52] member of a family of transcription factors frequently activated by cellular stress [62, 63] including ischemia (S2 Table). As shown in the gene schematic (Fig 2A), one isoform of Nr4a3 is about 40 kbp in length; however, short isoforms that terminate internally have been identified. Non-quantitative analysis of Pol II density using 1/200 of standard ChIP (Fig 2B) reveals a near absence of basal transcription in non-proximal regions (1949–39565 bp) that is informational given most of these primers are capable of single copy PCR. In contrast, unambiguous Pol II signal at the promoter (-1343, -331, -329 bp) strongly suggests PPP is preventing productive elongation. Importantly, promoter proximal Pol II shows increasing density for a period of time after receptor stimulation, but prior to release of polymerase into productive elongation (Fig 2B and Fig A in S3 Fig). This behavior demonstrates that pausing of Pol II, and not the increase in Pol II recruitment, remains rate limiting for a period of time following activation. One consequence of low basal activity is that early faint Pol II signals are not hidden by signal from basal transcription. For most primers, faint Pol II density precedes the dominant wave of transcription (Fig 2B), but is lost upon five-fold reduction of ChIP input DNA (Fig A in S3 Fig **).** A very unusual aspect of Nr4a3 behavior is the failure of primers immediately downstream of the proximal pause position (79, 908, 1258 bp) to reflect proximally bound polymerase (Fig 2B and Fig A in S3 Fig). One explanation is a sonication-induced DNA break that obligatorily occurs on the 3’ side of the paused Pol II at a vulnerable point near the polymerase. Potentially related was the unusual behavior of the primer sets upstream (-2017 bp) and downstream (79 bp) of the paused Pol II. These primer sets produced inexplicable patterns relative to close neighboring primers that are starkly apparent with lower ChIP input (Fig 2B and Fig A in S3 Fig), and also failed to effectively amplify genomic DNA (Fig A in S3 Fig). A likely interpretation, particularly given the single sided cleavage associated with the Nr4a3 promoter region, is that restructuring of promoter proximal complexes upon activation leads to changes in signal due to altered sonication-induced cleavage.

Quantitatively, the near absence of basal Nr4a3 transcription in Fig 2B suggests Pol II density is less than 0.04% (<1 copy) and almost certainly less than <0.01%, as product is produced in less than a quarter of the reactions prior to gene activation. Indeed, even this signal may be a consequence of non-specific precipitation, as some background is inevitable in ChIP experiments. If the signal were entirely due to non-specific background it places a lower limit on the dynamic range of the Pol II ChIP assay (i.e. ~0.01%). Analysis of Nr4a3 activation with 5-fold less ChIP DNA brought activated signal within the dynamic range of the standard curve and still showed relatively even density along the gene (Fig A in S3 Fig). However, regression analysis revealed a statistically significant decrease in more distal regions (Fig B in S1 Fig) that is readily apparent in a comparative time course (Fig B in S2 Fig). Additionally, Pol II density in the first part of the gene was statistically higher (5.5%) by Student’s t-test than later in the gene where density was 3.3% (p<0.0025). The large increase in Pol II density associated with synthesis of the sequence common to both transcripts (>550-fold), appears to account for nearly all of the observed increase in mRNA (1,380-fold) reported by qPCR analysis of the combined signal (Fig 2C and 2E), indicating increased mRNA stability plays little role in Nr4a3 upregulation.

### Matrix ChIP analysis of Pol II density on the Nr4a3 supports ChIP results

As additional support for the Pol II ChIP methodology, we wished to compare our results to an alternative Pol II ChIP procedure. To this end, we adapted the high throughput Matrix ChIP procedure [49] and employed the anti-Pol II antibody, 4H8, to precipitate Pol II from extracts produced by our protocol. The 4H8 monoclonal antibody was raised against a CTD peptide containing 10 heptapeptide repeats phosphorylated at Ser5, but appears to bind both unphosphorylated and phosphorylated forms of the CTD. Perhaps because our ChIP extracts used detergents that were not optimized for the Matrix ChIP system, high background signal was observed in untreated samples limiting the dynamic range available for analysis. Nevertheless, matrix ChIP analysis employing qPCR at 7 primer locations along Nr4a3 (Fig 2F), revealed Pol II behavior essentially identical to that of standard ChIP including promoter associated increases in density at 3 and 5 minutes (-102, 497 bp), entry into productive elongation by 7 min (5166 bp) and the absence of significant distal density at 10 min (25598 bp). Matrix ChIP estimates of Pol II density also showed a reduction in distal Pol II signal (Fig 2F, 30 and 60 min.) that was similar to that of reported by standard ChIP. The apparent decrease in distal density observed by both assays could reflect pS5 dephosphorylation; however, an actual decrease in Pol II density seemed more likely, given the proximal and distal regions are separated by an internal polyadenylation site.

In light of lower levels of distal Pol II, it is noteworthy that ChIP data (Fig 2B) provides modest evidence of a very faint Pol II signature extending across most of the Nr4a3 gene at 7 min (18743, 24008 and 27993 bp) that is blunted at 10 min (18743 bp). Concordant with this failure of transcription to extend beyond mid-gene at 10 minutes, microarray analysis detecting only the long polyadenylated isoform actually reports a drop in message levels at 15 minutes, in contrast to qPCR employing upstream primers common to the short and long isoforms (Fig 2C). Further, qPCR employing these common upstream primers showed concordant increases (Fig 2D) whether analysis used cDNA primed with random hexamers (pre-mRNA + mRNA) or oligo-dT (only mRNA), demonstrating expression of a polyadenylated short isoform soon after arrival of Pol II. Attempts to use qPCR to quantitate the fraction of message diverted into a short isoform were unsuccessful, as analysis of low basal message was too variable to establish a modest 2-fold difference in relative activation. Nevertheless, these data demonstrate elevated transcription early in the Nr4a3 gene is associated with polyadenylation of the short isoform and suggests this polyadenylation site delays transcription of the long isoform.

### Initial transcriptional velocity on the Nr4a3 gene is rapid

Despite the lack of significant Nr4a3 expression prior to α_1a_AR stimulation, initial transcriptional velocity appeared to be very rapid. The first polymerases exit the promoter proximal region within 3 to 5 minutes and reach the distal end of the gene within 15 minutes (Fig 2B). Analysis of the dominant wave of transcription showed a very similar profile, as Pol II leaves the promoter around 5 minutes and reaches 34842 bp by 15 minutes (Fig 2B and Fig A in S3 Fig). Although precise estimation of velocity is problematic due to the length of the time windows, reasonable interpretation indicates a velocity at or above 3,500 bp/minute, even though the gene was essentially inactive prior to receptor stimulation. Singh and Padgett have used an application of qPCR to show that Pol II can transcribe very long genes at velocities near 4 kbp/minute [46]. Because PCR analysis of cDNAs avoids inherent limitations associated with Pol II ChIP (i.e. large DNA fragment size and promoter proximal signal), randomly primed cDNA derived from the same RNA employed for microarray and qPCR analysis, was used to analyze Nr4a3 transcriptional velocity with Pol II ChIP primers. Focusing on the initial rise in product, the first significant amount of pre-mRNA (Fig B in S3 Fig) was observed concurrent with Pol II exit from the promoter proximal region at about 5 minutes, and later as Pol II traversed much of the gene within 15 minutes (27993, 34842 bp). Agreement between arrival of the main wave of Pol II (Fig A in S3 Fig) and the initial increase in pre-mRNA (Fig B in S3 Fig) is striking and again suggests transcriptional velocity of at least 3,500 bp/minute. Because basal activity is absent, the initial increase in pre-mRNAs is not due to changes in RNA stability and neither is there any signal from genomic DNA contamination.

### Rapid activation of Nr4a1 without a prepositioned polymerase

**Fig 3:**
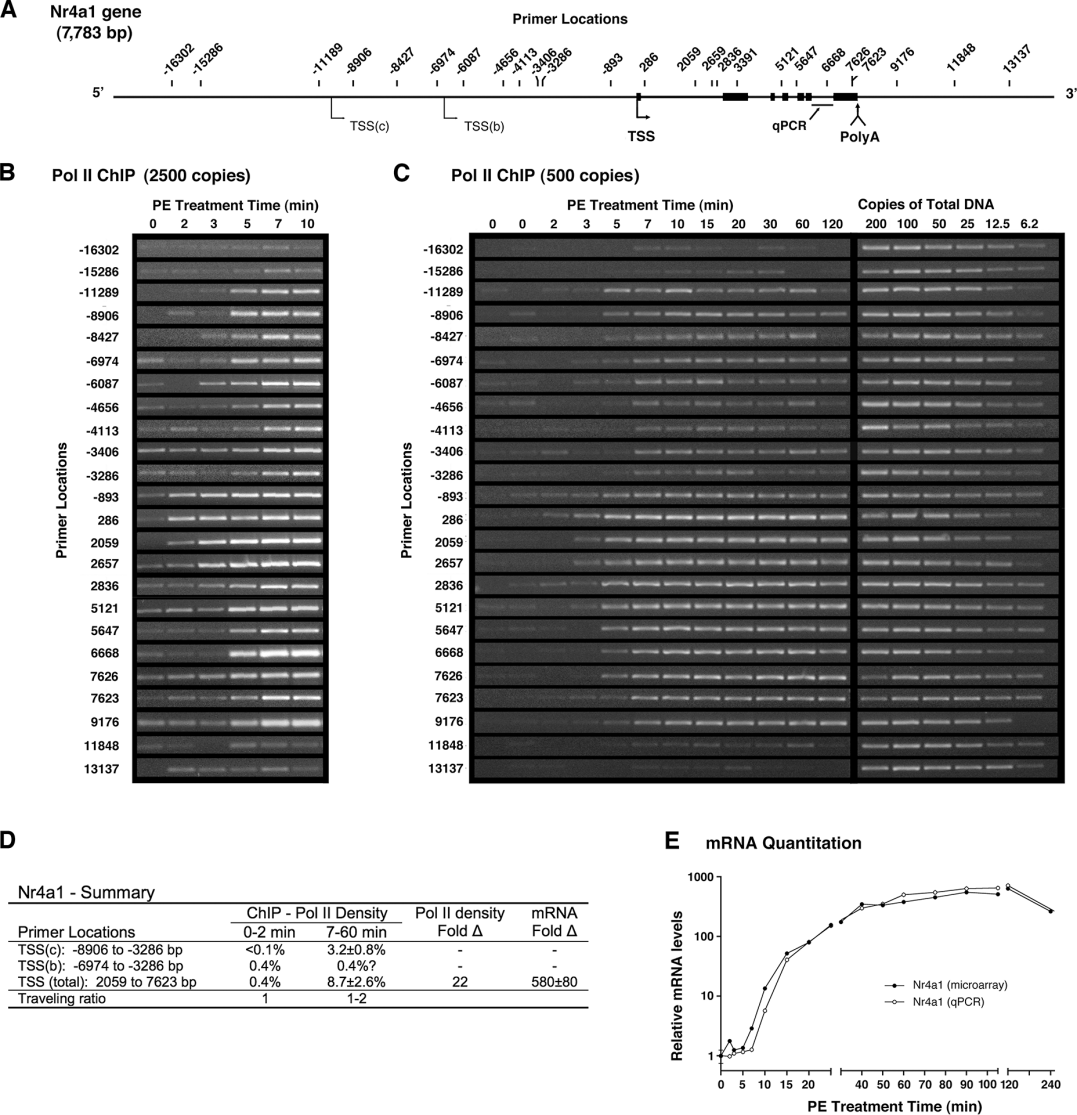
Temporal Pol II ChIP analysis of Nr4a1 shows a distinct basal and two activated mRNAs. (**A**) Schematic of Nr4a1 gene including upstream TSSs. Primer locations are indicated relative to the dominant TSS. (**B**) Non-quantitative Pol II ChIP with high input DNA (2500 genome copies) suggests most basal transcriptional activity originates from the upstream TTS(b) location. Activated Pol II density shows signal saturation downstream of both the dominant TSS and second activated TSS(c). (**C**) Quantitative Pol II ChIP using input DNA from 500 genome copies. Pol II density from activated TSS(c) is within dynamic range. (**D**) Summary of transcriptional activity. Pol II density expressed as percent precipitation efficiency. TaqMan qPCR primer set used to quantitate Fold-Δ in total mRNA (shown in next panel). (**E**) Analysis of relative Nr4a1 mRNA levels. Agilent microarray analysis (●) of polyadenylated mRNA and TaqMan qPCR analysis (○) of total mRNA (mRNA + pre-mRNA) following excision of the terminal intron.

A second member of the Nr4a family of stress response genes, Nr4a1, is also strongly upregulated by cardiac ischemia and α_1a_AR stimulation (S2 Table). Following agonist addition, Pol II ChIP revealed complex transcriptional behavior requiring many primer pairs at the positions indicated in the Nr4a1 gene schematic (Fig 3A). Non-quantitative analysis using 1/200 of standard ChIP (Fig 3B), shows no basal concentration of Pol II near any transcription start site, but does show modest levels of basal transcription, most of which appears to originate from a putative TSS(b) upstream of the dominant TSS (Fig 3A). Consistent with the position of TSS(b), this region is near the start site for a recently referenced rat gene (XM_006242358.2) and orthologous to conserved sequence containing a putative human TSS (i.e. NM_173158.1). At this ChIP input, signal saturation was apparent within 7 minutes of receptor stimulation on the dominant transcriptional unit and 11 kbp upstream (Fig 3B) near the location where the recent genome build [Rnor_6.0] annotates a TSS (-11644 bp). However, ChIP analysis using 5-fold less input DNA (Fig 3C) showed upstream signal to be less than that associated with the dominant TSS, which continues to display saturating signal (-893 to 9176 bp).

Despite its complexity, the image of Nr4a1 gene activation in Fig 3C provides an interpretable contour map of transcriptional activity. Between primers displaying little signal before (-16302, -15286 bp) and after (11848, 13137 bp) the transcribed regions, the two distinct areas of activated transcription are obvious. Although no TSS initially exhibits PPP, Pol II density proximal to the dominant TSS (-893, 286) increased prior to the start of productive elongation and both activated promoters appear to display slightly elevated density. Activation of the dominant genetic unit is rapid, as Pol II exits the promoter proximal regions by 3 min (2059, 2657 bp) and reaches the end of the gene by 5 min (7626 bp). Relative to the dominant start site, the upstream TSS(c) initiates transcription later and downregulates sooner. Over unique upstream sequence, transcription initiated from TSS(c) appears rapid and presumably continues to the common polyadenylation site. The image also suggests sustained transcription from TSS(b) consistent with faint proximal signals at 3 and 5 minutes (-6974, -6087 bp).

Quantitatively, basal Nr4a1 transcription originating largely from TSS(b) displayed Pol II signal below the quantitation curve across the gene (Fig 3C); however, nearly all primers produced signal. Specifically, 28/32 unstimulated points produced detectable product, suggesting a Poisson derived value of ~2 copies/reaction (ChIP efficiency of ~0.4%), consistent with the disappearance of most basal signal from an analysis using 2.5-fold less input ChIP DNA (S4 Fig). At this lowest input, quantitation across the dominant transcriptional unit showed similar Pol II densities and yielded an average value of 8.7% (Fig 3D and S4 Fig). Across the coding regions of Nr4a1, comparison of basal and activated Pol II densities suggest transcriptional upregulation of 22-fold (Fig 3D). Given the activated Pol II density attributable to the upstream TSS(c) was 3.2% (from Fig 3C), this initiation site appears to be responsible for about one third of the total message synthesis (Fig 3D). Consistent with transcriptional completion by the earliest polymerases around 5 minutes, microarray analysis shows polyadenylated mRNA levels rising by 7 minutes slightly ahead of terminal splice site removal reported by qPCR (Fig 3E). Quantitatively, qPCR was in agreement with microarray data and reported a 560-fold increase in mRNA levels (Fig 3D). In combination with the 22-fold increase in transcription suggested by increased Pol II density, this value implies a 25-fold increase in message stability and a major role for mRNA stabilization in Nr4a1 activation.

### Transcriptional velocity on Nfil3 is rapid but expression is delayed

**Fig 4:**
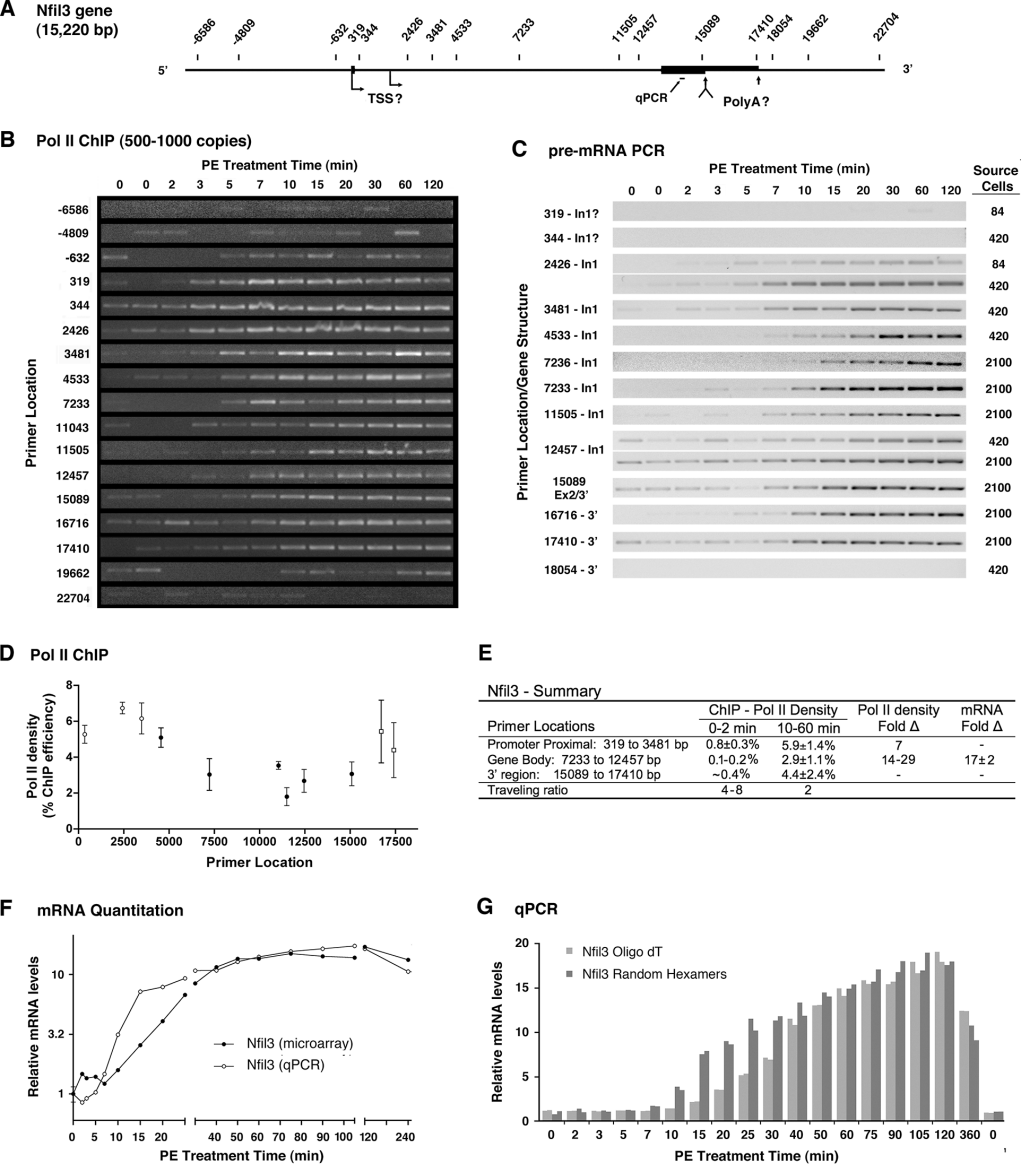
Analysis of Nfil3 activation shows rapid transcription but delayed mRNA maturation. (**A**) Schematic of Nfil3 gene showing primer locations relative to the annotated TSS. Putative TSS and PolyA sites also shown. (**B**) Quantitative Pol II ChIP experiment using input DNA equivalent to 500–1000 genome copies. Profiles are representative (n = 3–5) except for primer at 319 (n = 1). (**C**) Qualitative PCR analysis of pre-mRNA expression using Nfil3 ChIP primers. The number of source cells used to produce cDNA for each profile is shown at the far left. 2,100 cells corresponds to input cDNA from 12.5 ng of total RNA. Negative images are presented to distinguish PCR analysis of pre-mRNAs from Pol II ChIP data. (**D**) Accumulated Pol II ChIP data from 2 to 4 independent measurements. Error bars indicate SEM, except for points with only two values (thicker error bars indicate the difference from average). (**E**) Summary of transcriptional activity. Pol II density expressed as percent precipitation efficiency. TaqMan qPCR primer set used to quantitate Fold-Δ in total mRNA. (**F**) Analysis of mRNA levels. Agilent microarray analysis (●) of polyadenylated mRNA and TaqMan qPCR analysis (○) of total mRNA (mRNA + pre-mRNA) directed at terminal exon sequence. (**G**) Comparative TaqMan qPCR analysis of oligo dT and random hexamer primed cDNA. Profiles shows early pre-mRNA synthesis relative to delayed formation of the polyadenylated mRNA. Replicated data presented on a linear Y-axis to accent the temporal divergence.

E4bp4 protein (Nfil3) is a transcription factor encoded by a gene annotated as 15 kbp in length (Fig 4A) and involved in stress and immune responses as well as circadian rhythms [64]; all processes that may be regulated by α_1a_ARs. Despite complications, analysis of Nfil3 activation revealed high transcription velocity coupled with cotranscriptional regulation that delayed expression of mature message. Problematically, Pol II ChIP showed both basal and activated density shifted downstream relative to the expected TSS (Fig 4B), with the later shown by accumulated measurements for each primer set (Fig 4D). Subsequent analysis of pre-mRNA expression using ChIP primers demonstrated a nearly complete absence of pre-RNA synthesis immediately downstream of the expected start site (Fig 4C: 319, 344 bp). However, a recently predicted mRNA variant (X1) initiates downstream (2183 bp) and could explain both the low level of upstream Pol II (-614 bp) and the range of proximal density common to basal and activated Pol II (319–3481 bp). Both increased Pol II (Fig 4B) and pre-mRNA (Fig 4C) suggest promoter escape by 5 minutes and arrival at the 3’ terminus by 7 minutes, consistent with qPCR analysis directed at sequence within the final exon that also shows a small increase at 7 minutes (Fig 4F). Together, these results demonstrate the initial wave of Pol II displayed rapid transcriptional velocity near 4,000 bp/min. Further complicating analysis, none of the 3’ primer pairs (15089, 17410, 18054 bp) were part of the annotated Nfil3 mRNA, nevertheless, expression of these target sequences was robust (Fig 4C) and the profiles matched qPCR analysis directed at the terminal exon (Fig 4F), suggesting these sequences have been incorporated into mature messages. Of interest, pre-mRNA analysis (Fig 4C) showed a faint “pre” wave of transcription, which was proximal at 2 minutes (2426, 3481 bp) and more distal at 3 minutes (7233–17410 bp) followed in each case by a reduction in pre-mRNA levels.

Quantitative analysis of Nfil3 shows upregulation was a result of increased transcription related to Pol II recruitment and partial abrogation of pausing. In proximal regions, Pol II density before activation (0–2 min) was 0.80% as compared to a density of 6% during the plateau of activated expression, clearly demonstrating a substantial increase in recruitment (Fig 4E). Basal Pol II density near the center of the gene was below the lowest quantitation point containing 3.1 genomic copies (0.3% ChIP efficiency); however, signal was usually present suggesting a density of 0.1% to 0.2% (Fig 4E). A modest if partial reduction in pausing is suggested by the drop in the traveling ratio from 4–8 to 2 following gene activation (Fig 4D and 4E). As Pol II density near the center of the Nfil3 gene increased 14- to 29-fold following activation, increased transcription is ultimately responsible for the 18-fold increase measured by qPCR (Fig 4E).

### Early transcription of the Nfil3 gene leads to pre-mRNA degradation

Following Nfil3 activation, increased Pol II density appears to be associated with the region near the annotated polyadenylation site (Fig 4B and 4D). Although activated Pol II density in the 3’ region was not statistically higher, this pattern was apparent in individual experiments (data not shown) and is also present under basal conditions, suggesting a pause associated with 3’ end formation as has been reported for some genes [65, 66]. Importantly, this 3’ pause was associated with failure of microarray analysis to detect a substantive increase in polyadenylated mRNA at 7 or even 10 minutes despite the fact that qPCR (Fig 4F), a well as Pol II ChIP (Fig 4B) and pre-mRNA PCR (Fig 4C), show increased transcription of distal sequence. Evidence suggests weak polyadenylation sites can induce pre-mRNA degradation [18, 19], thus it was possible that the inefficient polyadenylation at the annotated site led to degradation of nascent pre-mRNA. To confirm the discrepancy between pre-mRNA and polyadenylated mRNA with a single technology, we used qPCR to analyze cDNA synthesized using random hexamer or oligo-dT primers to compare expression of total mRNA (pre-mRNA+mRNA) to polyadenylated mRNA (Fig 4F). Use of a linear y-axis scale makes it clear that production of polyadenylated mRNA remains consistently below that observed with random priming from 10 to 30 minutes. Given that new transcription continues to be initiated (Fig 4B), this failure to increase polyA mRNA implies pre-mRNA degradation, particularly since the time gap observed between pre-mRNA synthesis and polyadenylation (Fig 4G) is long (>10 minutes) relative to the times (~min) required for degradation [18, 19]. It should be recognized that demonstrating the transient existence of an unstable message is non-trivial [17], and that incompatible measurements of the pre-mRNA and polyadenylated mRNA may be the best available indicator of degradation. Of obvious importance to Nfil3 expression, 30 to 40 minutes after gene activation total RNA and polyadenylated mRNA equalized (Fig 4F and 4G), suggesting nascent pre-mRNA degradation was no longer regulating induced expression. Potentially consistent with a coordinated process linking initiation to termination, Nfil3 was the only gene that showed increased Pol II density at 30 and 60 minutes (Fig E in S2 Fig).

### Transcription of the Dusp5 gene is rapid

**Fig 5:**
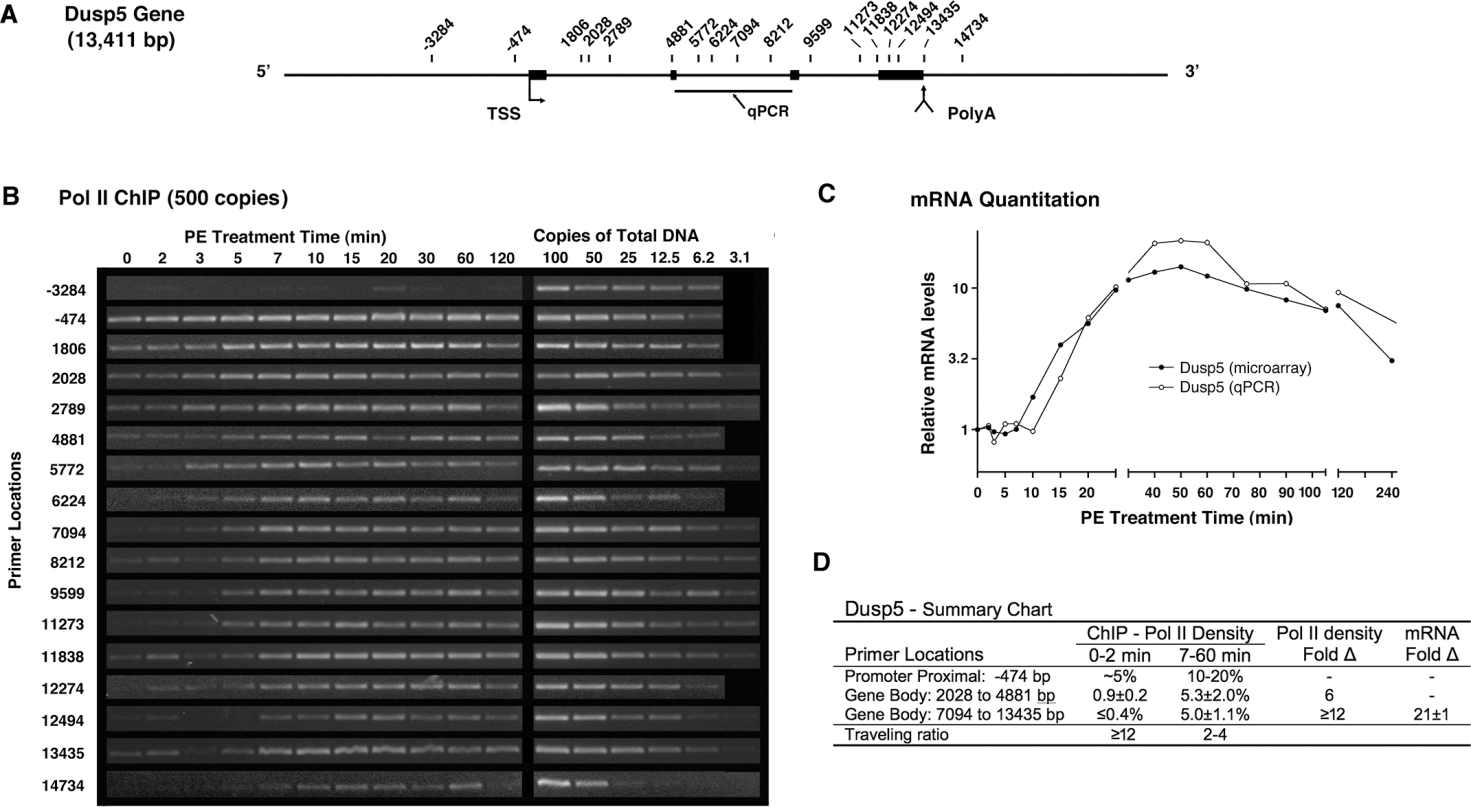
Pol II ChIP analysis of Dusp5 activation shows rapid transcription. (**A**) Schematic of Dusp5 gene showing primer locations relative to the annotated TSS. (**B**) Quantitative Pol II ChIP experiment using input DNA equivalent to 500 genome copies. Profiles are representative (n = 2–3). (**C**) Analysis of Dusp5 mRNA levels. Agilent microarray analysis (●) of polyadenylated mRNA and TaqMan qPCR analysis (○) of total mRNA (mRNA + pre-mRNA). (**D**) Summary of transcriptional activity. Pol II density expressed as percent precipitation efficiency. TaqMan qPCR primer set used to quantitate Fold-Δ in total mRNA.

Another relatively long IEG gene, Dusp5, is about 13 kbp in length (Fig 5A) and codes for a nuclear phosphatase that inactivates MAPKs [67], presumably generating feedback inhibition of α_1a_AR-induced activation. ChIP analysis (Fig 5B) shows Pol II exited the promoter proximal region of Dusp5 between 2 and 3 minutes (2789, 4881 bp) and traversed most of the gene by 5 min (9599, 11273 bp), consistent with a transcriptional velocity of ~4,000 bp/min. Transcription reached an activated plateau by 7 minutes (Fig 5B) during which Pol II density was statistically constant across the gene (Fig D in S1 Fig) and at time points from 7 to 60 minutes (Fig D in S2 Fig). Activation of the Dusp5 gene appears to involve increased recruitment and partial abrogation of pausing; however, for unknown reasons basal density downstream (2028–4881 bp) of the expected proximal region was higher than at more distal locations (Fig 5D). The low signal on the distal body of the gene was usually present but generally below the lowest standard (3.1 copies) and presumably represented 0.2–0.4% ChIP efficiency (1–2 copies). Comparison of this crude value to activated expression levels (5%), suggested an increase in transcription of >12-fold, which could account for the 21-fold increase in mRNA measured by qPCR (Fig 5C and 5D). Given the uncertainties, efforts to more accurately establish the relative contributions of recruitment, pausing and increased mRNA stability were not pursued.

### Upregulation of Gprc5a is delayed by an intragenic transcriptional pause

**Fig 6:**
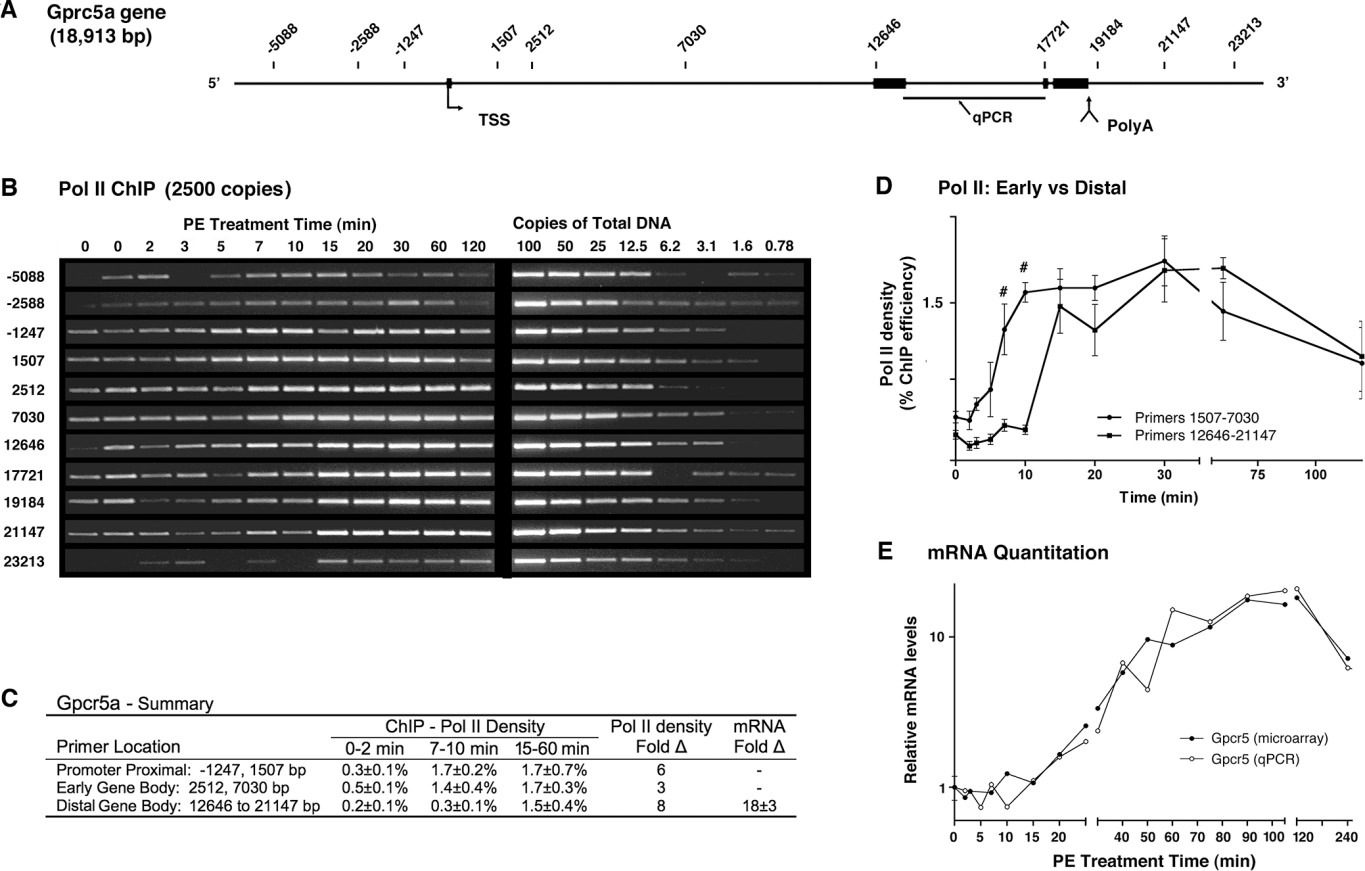
Temporal Pol II ChIP analysis of Gprc5a activation reveals a transcriptional pause. (**A**) Schematic of Gprc5a gene showing ChIP primer locations relative to the annotated TSS. (**B**) Quantitative Pol II ChIP experiment using input DNA equivalent to 2500 genome copies. Profiles are representative (n = 2–3). (**C**) Summary of transcriptional activity. Pol II density expressed as percent precipitation efficiency. TaqMan qPCR primer set used to quantitate Fold-Δ in total mRNA. (**D**) Temporal comparison of Pol II density on the early and distal parts of the Gpcr5a gene. The profiles are statistically different by two-way ANOVA and the Bonferroni post-test identifies statistical difference at 7 (p<0.01) and 10 (p<0.001) minutes (indicated by #). (**E**) Analysis of Gpcr5a mRNA levels. Agilent microarray analysis (●) of polyadenylated mRNA and TaqMan qPCR analysis (○) of total mRNA (mRNA + pre-mRNA). Microarray directed at sequence within the second exon.

The orphan G protein coupled receptor, Gprc5a, is also upregulated by cellular exposure to retinoic acid and may inhibit cell proliferation [68], potentially suggesting a role in the antiproliferative phenotype of the α_1a_AR. The promoter proximal region of Gprc5a gene (Fig 6A) shows increased Pol II density within 3 and 5 minutes of receptor stimulation prior to polymerase escape between 5 and 7 minutes (Fig 6B). Although the dominant wave of Pol II traverses ~30% of the gene by 7 min, density in distal regions (12646–21147 bp) increased only modestly prior to 15 min (Fig 6B and 6C). Comparing the time dependence of increasing Pol II density between early and distal regions of the gene revealed a substantial early gap that is abrogated by 15 minutes (Fig 6D). Consistent with a transcriptional block near the center of the Gprc5a gene, basal density was also elevated in the early gene body (Fig 6C). The functional significance of intragenic transcriptional blockade is supported by the failure of early transcription to produce significant amounts of pre-mRNA (qPCR) or polyadenylated mRNA (microarray) until after the internal block disappears (Fig 6E). In addition, the failure of Pol II that evades blockade to substantially increase polyadenylated message prior to 20 minutes suggests transcriptional events were linked to slow polyadenylation. Quantitatively, Pol II density in distal regions before and after activation produced respective ChIP efficiencies of 0.2±0.6% and 1.5±0.4% (Fig 6C), indicating an 8-fold increase in transcription that accounts for much of the 18-fold increase in mRNA (Fig 6E), but may also suggest a second mechanism.

## Discussion

**Fig 7:**
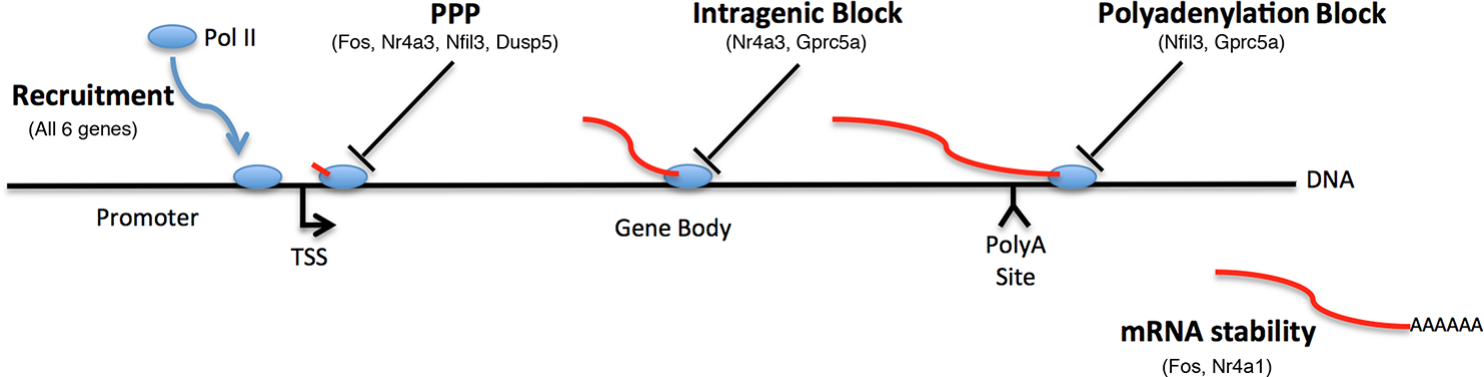
Schematic illustrating the dominant regulatory events that control IEG expression. Together, Pol II ChIP and mRNA expression analysis can determine both the temporal effect and relative contribution of major transcriptional events to gene activation, including Pol II recruitment, promoter proximal pausing, internal and polyadenylation associated transcriptional blocks and control of mRNA stability. Gene names indicate events regulated during activation of the indicated gene.

Based on a limited set of genes, analysis of IEG expression using Pol II ChIP and mRNA analysis suggests gene regulation will often involve both recruitment and post-recruitment mechanisms. This analysis was able to distinguish increased recruitment of Pol II and regulation of mRNA stability from co-transcriptionally regulated mechanisms including: abrogation of promoter proximal pausing, intragenic transcriptional blocks, and delayed mRNA maturation due to polyadenylation associated mRNA degradation (Fig 7). Even though co-transcriptional regulatory mechanisms were present for 5/6 of the genes analyzed, increased recruitment was a substantial factor contributing to increased mRNA levels for all six genes. Consequently, simple release of lower levels of basal Pol II from promoter proximal regions could not have induced full gene activation. Techniques for measuring genetic mechanisms (Fig 7) are generally performed under different assay conditions and are not quantitatively comparable, thus it is difficult to use these methods to infer the relative importance of the individual mechanisms. As this information is essential to delineating the role of specific cell signaling pathways in activating particular genetic mechanisms, current understanding of the process employed by these signaling pathways to integrate genetic mechanisms is necessarily incomplete. For IEG activation during severe stress, the time required to complete the transcriptional process is also important, as cells must race to produce mature mRNAs in the face of deteriorating energetic situations [69] and translational shutdown [69, 70]. Integrated temporal analysis simultaneously addresses the relative importance of major mechanisms of gene regulation (Fig 7) both temporally and quantitatively. Although long IEG genes could possess unusual characteristics, temporal analysis of these six genes displayed a broad range of regulatory mechanisms that may be of general relevance.

Conceptually, it is clear that Pol II ChIP can measure polymerase density at any location along a gene as transcriptional activation proceeds [56]. Nevertheless, difficulties inherent to the procedure have presumably prevented widespread application to temporal dissection of mammalian gene activation. Beyond the technical challenges, fundamental assumptions have not been established and one of these, random DNA cleavage, violates the intuitive view that single strand DNA created by protein association is more likely to produce sonication induced breaks. Consistent with this idea, biological context has been shown to influence sonication-induced DNA cleavage [57]. As described above, we observe ChIP signals associated with pausing of Nr4a3 that strongly suggest non-random DNA cleavage has impacted apparent Pol II density. In addition, ChIP primer sets that produce products that cross the promoter proximal region were avoided as such primers performed badly in the past (e.g. PPIA and GAPDH in [48]), we suspect due to DNA cleavage near the paused polymerase that destroyed the sequence necessary for PCR.

The presence of paused promoter proximal polymerase at some human [71, 72] and many drosophila [73] genes has been recognized for decades; however, only recently has the generality of PPP been unequivocally established in mammals [10–15]. Quite problematically for mammalian studies, Pol II phosphorylation patterns detected by Pol II ChIP are superficially similar to early observations in yeast, which suggested distal pS5 dephosphorylation. These early studies employed 3 monoclonal antibodies with specificities to different CTD phosphorylations in part due to their high affinities [48] presumably resulting from multisite binding to the repetitive CTD [74]. These antibodies were the IgM class H14 that requires CTD phosphorylation at S5 (pS2 permissive), the IgG class, 8WG16, which binds the unphosphorylated CTD (pS5 but not pS2 permissive), and another IgM class antibody, H5, which has more affinity for pS2 than pS5 but prefers doubly phosphorylated repeats [75, 76]. Because early ChIP studies in yeast also employed effective non-CTD antibodies that reported constant Pol II densities across genes [55, 75, 77], the disappearance of H14 detectable signal as Pol II traveled distally was interpreted as pS5 dephosphorylation [75, 77]. However, more recent higher efficiency Pol II ChIP experiments with pS5 specific antibodies have demonstrated a more even distribution of polymerase across yeast genes, implying pS5 is present during elongation [78–81]. Surprisingly, in both yeast [75] and mammals [48], the 8WG16 and H5 antibodies display reciprocal patterns of Pol II precipitation, suggesting either rapid S2 phosphorylation that prevents recognition by the 8WG16 antibody, or a distinctly different CTD conformation cooperatively induced by the presence of nonsaturating pS2. Although, other CTD antibodies are now available [78, 82], the specificity of these antibodies is often poorly documented.

In contrast to yeast, analysis of Pol II density on mammalian genes has always shown pS5 to be maintained in distal regions of these much longer genes. While early Pol II ChIP studies in human HeLa and LNCaP cells using the H14 (pS5) antibody did show higher Pol II density at the 5’ end of genes, concurrent analysis with Pol II N20 (total polymerase) produced a very similar pattern, demonstrating phosphorylation was maintained [48, 83]. Concordance between Pol II density patterns reported by ChIP analysis with the N20 and H14 antibodies has also been observed in others mammalian models [17, 59, 84–87]. In agreement with these results, a newer pS5 specific monoclonal antibody (3E8) produced the same profile as total polymerase across the T cell receptor beta gene [82]. Thus, in controlled studies, the ability of H14-based ChIP to accurately report the density of mammalian Pol II is better established than is the case for most CTD antibodies, at least through the polyadenylation signal.

The Pol II density near the promoters of unstimulated Nr4a3, Fos, Dusp5 and Nfil3 genes was almost certainly due to PPP and the most common co-transcriptional mechanism of activation involved partial or full abrogation of basal PPP. Although proximal Pol II pausing is reported as a single value, the mechanistic details of pausing remain unclear and may involve diverse modes of action; however, these issues are beyond the scope of the current study. Among the genes studied here, only Fos showed complete abrogation of pausing. Importantly, uniform density across the activated Fos gene would not have been observed if significant amounts of anti-sense transcription were producing signal in the Pol II ChIP assay. Very recently, Ahrner and coworkers performed genome wide time courses of induced transcription start site usage in 33 mammalian cell models following multiple stimuli [88]. Even though their methods should have detected capped anti-sense transcripts, none of the six genes analyzed in the present study produced significant anti-sense signal, providing a strong indication our results have not been impacted by this phenomenon. For Fos, the 2.4-fold decrease in traveling ratio could have provided only a fraction of the 170-fold increase in maximal message levels. In contrast, PPP on Nr4a3 was essentially complete prior to activation and for about 5 minutes thereafter. Even though abrogation of pausing was a dominant rate-limiting mechanism in Nr4a3 activation, increased recruitment contributed substantially to increased expression. Activation of the Dusp5 and Nfil3 also appears to involve partial abrogation of pausing. On the other hand, PPP was not apparent on the Nr4a1 and Gprc5a genes under basal conditions, unambiguously demonstrating release of a prepositioned polymerase did not contribute to gene activation. Although abrogation of PPP was a common mechanism of activation, elongation was temporally separable from Pol II recruitment on the Nr4a3, Nr4a1 and Nfil3 genes, as proximal Pol II density increased prior to polymerase exit from the promoter region. This apparent PPP-induced delay in productive elongation supports the concept that pausing ensures a properly assembled elongation complex [89]. Although we have not focused on acute “pre” waves of transcription as apparent in the Nfil3 pre-mRNA profile, the reduction in transcription at the time point following the initial wave also suggests a fidelity mechanism associated with pausing. For most genes a minor increase in the speed of expression due to Pol II “prepositioning” seems of modest biological significance; however, the need to maintain fidelity in control of expression is obviously universal.

Given the established role of mRNA stability in the activation of immediate early genes, the increased mRNA stability observed for Fos and Nr4a1 was not unexpected. In this report increased mRNA stability was deduced from the difference between transcriptional upregulation (Pol II ChIP) and increased mRNA levels, an approach that despite caveats has the advantage of implicit use of a single assay condition. As applied here, this approach requires reliable Pol II ChIP and mRNA quantitations and cannot dependably establish small changes (<2-fold) in mRNA stability, as may have occurred for Dusp5. In addition, the quantitative estimate is dependent on the assumption that basal transcriptional velocity is equal to activated velocity. Although we have not established this fact, basal transcription on many mammalian genes appears to be rapid [60, 61] and relatively minor differences within the range of 3 to 4 kb min would require modest correction to implied transcription rates based on increased Pol II density. Further, for Fos and Nr4a1, increased mRNA stability appears to be important for accelerating upregulation beyond that possible with transcription alone, as apparent Pol II densities of ~10% were close to that of the highly expressed GAPDH gene (e.g. ~15% see [48]), suggesting near maximal rate of transcription.

During studies elucidating the process of the 3’ end formation, the Proudfoot and Martinson laboratories recently discovered a novel paradigm in gene regulation involving polyadenylation associated pre-mRNA degradation [18, 19]. Earlier studies had shown that polyadenylation and transcription termination were linked [90, 91] and accompanied by transcriptional pausing [92, 93] associated with CPSF complex binding to the newly synthesized hexanucleotide polyadenylation signal of the pre-mRNA [93–95]. Although cleavage and polyadenylation at efficient sites can take place within 10 to 20 seconds [96], cleavage at inefficient sites is not only delayed [93], but is also associated with much lower production of mature mRNA [97]. Concordantly, decreased transcriptional velocity associated with 3’ pausing has now been shown to induce PolyA-dependent degradation of pre-mRNA when 3’ cleavage and polyadenylation is delayed even by a minute [18, 19]. Together, these studies strongly suggest polyadenylation plays a direct role in gene regulation, in addition to the indirect effects that alternative polyadenylation has on mRNA stability [98]. Our analysis of both Nfil3 and Gprc5a imply polyadenylation-associated degradation, given that the dominant wave of Pol II transcribes through the annotated PolyA site without producing a substantive and concordant increase in polyadenylated message. For Nfil3, the lag of nearly 10 minutes observed before polyadenylated mRNAs increases to the level of the pre-mRNAs, suggests an extended period of pre-mRNA degradation that is eventually overcome. Importantly, this transience also implies a secondary mechanism of gene activation that is engaged about 30 minutes after receptor stimulation. Of potential relevance, a recent study suggests that high polymerase densities in the 3’ region is uncommon [66], suggesting strong but infrequent 3’ pausing of Pol II may indicate polyadenylation associated pre-mRNA degradation.

Distinct from the other genes, increased mRNA accumulation of the Gprc5 gene appeared to be delayed as a consequence of slower transcriptional progression, similar to behavior reported for the PUMA gene [17]. Pol II ChIP suggested the delay resulted from an intragenic transcriptional block that was subsequently abrogated, leading to an even distribution of Pol II across the activated gene. As the intragenic blockade on Gprc5a is also present under basal conditions, abrogation appears to have contributed to the transcriptional response. Of note, this phenomenon may be rare, as intragenic blockade has not been reported in multiple types of “-omic” studies where it should have been apparent.

*A priori*, the theory that IEGs are short to enable rapid transcription is compelling, while the temporal pattern of IEG activation and expression obviously controls the relative amounts of individual transcripts as a stress response unfolds. Consequently, the time required for transcriptional events during mRNA maturation is an element of IEG regulation whether or not any particular mechanism impacts maximal or homeostatic expression levels. Rapid activation occurred for all of the genes studied here, as Pol II recruitment increased on Nr4a1 by 2 minutes and on Fos, Nr4a3, Dusp5, Nfil3, Icer and Gprc5a by 3 to 5 minutes. Indeed, during Fos activation most transcriptional events leading to mRNA maturation occurred within a 2-minute window from 3 to 5 minutes after receptor stimulation. The fact that most IEG genes are short and rapidly activated also highlights the extent to which long IEG genes are atypical; suggesting a functional basis underlies their length. As described by Wada and coworkers, one functional consequence of longer genes is “… the time spent transcribing … long introns, … allow polymerases to convert space into time” [16]. Given the likelihood long IEG gene length is meant to delay expression, it may be unsurprising that genes such as Nfil3 and Gprc5a use additional mechanisms to control the time required for mRNA maturation.

Although some studies suggest that a range of transcriptional velocities occur in mammalian cells, transcription of IEGs induced by α_1a_AR stimulation was rapid and approached maximal observed rates [99, 100]. Pol II ChIP analysis of the Nr4a3 gene was particularly informative and reported transcriptional velocities at or above 3.5 kbp/minute. Although time windows were too widely spaced to unambiguously establish maximal velocities, analysis of at least two genes, Dusp5 and Nfil3, strongly suggested rates near 4 kbp/min. Indeed, with the exception of Gprc5a for which elongation was blocked, all of the genes analyzed here appear to transcribe very rapidly. Other studies have suggested rapid mammalian transcription including: 1) an early analysis of the 2,400 kbp dystrophin gene showing transcription of 2.4 kbp/min [101], 2) a comprehensive confocal-based kinetic analysis that suggested maximal transcriptional velocities of 4.3 kbp/min for a single polymerase [99] but implied much lower average velocities, 3) a recent H14 Pol II ChIP/tiled array analysis [16] of long genes activated by tumor necrosis factor-α, which transcribed at 3.1 kbp/min and 4) a compelling study of long genes by Singh and Padget, which demonstrated transcriptional velocities of ~4 kbp/min on long genes [46]. As has been noted [100], rapid transcription in the latter study could have resulted from artificial activation of previously transcribed genes which retained characteristic histone modifications often claimed to enable transcription. In contrast, our analysis of Nr4a3 provides evidence that transcription can proceed at rates at or above 3.5 kbp/min on essentially inactive genes subject to biologically relevant regulation.

More recently, “-omic” approaches have been used to simultaneously estimate transcriptional velocities on groups of genes, and while some report a range of velocities [102] others suggest transcription is usually around 3.5 to 4 kbp/minute [60, 61]. One study suggested velocities associated with upregulated transcription were lower at the beginning of gene expression but accelerated later in the gene [102]. While our Pol II ChIP data does not obviously support this view, 4/6 genes we analyzed trended toward higher densities at early time points (see S2 Fig), suggesting a tendency for the initial wave of transcription to pause more frequently. Such pausing would lead to somewhat lower average velocities, not necessarily apparent at the leading edge of transcription. Using natural receptor ligands, the same study found velocities were dependent on the gene, the activating signal and the level of gene expression [102]. Although the later observation could explain the high rates of transcription produced by α_1a_AR stimulation, the probability that IEGs associated with stress responses are rapidly transcribed seems high, given the need for acute expression and evidence supporting rapid transcription [46, 60, 61]. Indeed, low velocity estimates may be due to transient pausing not apparent in the widely spaced time points used in “-omic” analysis.

Evidence that Pol II density increases near splice junctions suggests a reduction in transcriptional velocity [103]; however, these modest increases in density correspond to very transient pauses that should have little impact on elongation velocity [60]. As our ChIP assay has fairly low resolution, it was unlikely we would detect such short duration effects. It has been established that polyadenylation is generally coupled to removal of the final intron [104, 105] and detailed mechanistic studies suggest polyadenylation proceeds excision of the final intron [60, 106–108], which may in turn license transcription termination [109]. Concordantly, we observed increased polyadenylated message slightly before (or concurrent with) excision of the terminal intron of Nr4a1 and several other genes (Morris, unpublished).

Phenotypically, α_1a_AR stimulation is associated with a hypertrophic response in nonproliferative myocytes [43] and an anti-proliferative hypertrophic response in most cellular models including rat-1 fibroblasts [37, 45, 50]. In rat-1 cells, high agonist concentrations induce a strong and partially sustained increase in intracellular calcium and p38 activation as well as transient Jnk activation; Erk however, is acutely inhibited (see [37] and references therein). These and other signaling pathways causally connected to α_1a_AR-induced gene activation [44] create a distinct biological phenotypic that is quite different from that of the α_1b_AR and α_1d_AR. This biologic divergence is particularly clear in the FANTOM analysis of start site usage for more than 1000 cell and tissue models (available on the ZENBU browser), which shows high α_1a_AR expression almost exclusively in differentiated human tissues (top 81/85). This in contrast to the other subtypes, whose expression is evenly distributed between proliferative and differentiated models [110]. While it seems remarkable that α_1a_AR stimulation in the rat-1 fibroblasts has qualitatively recapitulated much of the IEG pattern produced by other cardiomyocyte stress responses, quantitatively the responses are quite different (see S2 Table). Given the results presented above, we presume that varied contributions from multiple mechanisms will explain both similar and differential gene activation.

In mammals, IEGs are part of a complex interconnected set of signaling, transcriptional and translational responses designed to respond to stress, mitigate cellular injury and facilitate the removal of terminally damaged cells. In heart, Gq-coupled GPCRs including the α_1a_AR [111] act in concert with other signaling pathways to respond to ischemic and other stresses, producing protective [40, 41], preconditioning [32, 33] and hypertrophic [112] responses. Given the conservation of IEG expression across a wide range of cell types, these acutely expressed genes will almost certainly play a conserved if ill defined role in protecting the heart and other organ systems. As multi-level gene regulation appears common, determining the relative contributions of specific activating mechanisms may require integrated analysis for most genes. Eventually, integrated temporal analysis should enable investigation of the reciprocal interactions between these genetic mechanisms, protein expression and cell signaling, as regulatory cascades unfold into phenotypic responses.

## Supporting Information

S1 Fig Analysis of Pol II density across the transcriptional units following gene activation. ChIP density estimates of Pol II density during the indicated period of maximal, activated expression following α_1a_AR stimulation. Primer locations associated with 5’ end of upstream primer relative to the transcription start site (i.e. TSS bp is 1). (**A**) Pol II density on Fos, expressed as the number of precipitated DNA copies from a maximum of 200 copies, was constant across proximal and distal regions indicating a complete abrogation of proximal pausing. (**B**) Elevated promoter proximal Pol II density on Nr4a3 (500 copies max.) shows incomplete abrogation of pausing, while a distal decrease in density was reported by regression analysis as a negative slope (-0.28 copies/kbp) that was statistically significant (p = 0.022). Lower density in distal regions is a consequence of partial transcription termination at an internal polyadenylation site at 14,004 bp. (**C**) Pol II density across the dominant transcriptional unit of Nr4a1 (200 copies max) displays no systematic variation; however, the TSS appears to be associated with modest promoter proximal pausing. (**D**) For Dusp5 (500 copies max), elevated promoter proximal density even after activation suggests only partial abrogation of pausing. (**E**) Density across Gpcr5a shows no systematic variation in Pol II density following abrogation of an internal transcriptional pause. Because the values from each primer are not independent (due to use of a common quantitation curve), error bars show standard deviation. Linear regression analysis was done using the average value observed for each primer pair within the time period indicated.
(TIF)

S2 Fig Analysis of Pol II density at times following α_1a_AR stimulation. Combining quantifiable Pol II ChIP density estimates for many primers at each time point provides a measure of aggregate Pol II density on the gene. (**A**) Across the Fos gene, Pol II density, expressed as the number of precipitated DNA copies from a maximum of 200 possible copies, was constant between 7 and 60 minutes but showed decreased transcription at 120 minutes (n = 5). (**B**) For Nr4a3 (500 copies max), polymerase density (beyond promoter effects) was consistently higher upstream of the internal polyadenylation site at 14,004 bp; however, no significant temporal difference was observed in either proximal (n = 6–7) or distal (n = 8) regions [nonquantifiable time points were essentially zero]. (**C**) For Nr4a1 (200 copies max.), non-proximal Pol II density across the dominant transcriptional unit was constant from 7 to 120 minutes (n = 7). (**D**) Non-proximal density on Dusp5 (500 copies max.) was constant from 7 to 60 minutes but decreased by 120 minutes (n = 12). (**E**) For Nfil3 no significant difference in non-proximal Pol II densities (expressed as % ChIP efficiency) was found between 10 and 120 minutes (n = 15), nevertheless regression analysis showed an increase in slope (0.025±0.11% ChIP eff./min) that was statistically significant (p = 0.032) during the first hour following α1aAR stimulation. Individual density estimates for each point are independent as they were produced using different primers and quantititation curves. Consequently, linear regression analysis was performed using individual data values and the error bars show SEM. One-way ANOVA analysis with the Turkey post-test was used to demonstrate statistical difference and identify time points with non-maximal density (open circles). To avoid trivial statistical difference, early time points with basal polymerase density were not included in the statistical analysis.
(TIF)

S3 Fig Analysis of Nr4a3 activation by quantitative Pol II ChIP and qualitative PCR of pre-mRNA. (**A**) Quantitative Pol II ChIP using input DNA equivalent to 200 genome copies. Pol II density is within the dynamic range and quantitative estimates of ChIPed DNA copies (right) during the plateau of activated expression suggests density is modestly lower in more distal regions (14546–39565). Points not quantitated had inadequate reference curves (not determined; nd) or were above (≥) or below (<) the quantitation range. (**B**) Qualitative PCR analysis of Nr4a3 pre-mRNA synthesis using ChIP primers. Analysis of randomly-primed total RNA reveals initial pre-mRNA production corresponds very closely to arrival of the dominant wave of Pol II apparent in the prior panel. The analysis also shows the near absence of basal Nr4a3 transcription. The number of source cells used to produce cDNA for each profile is shown at the far left, with 2,100 cells corresponds to 12.5 ng of total RNA. Negative images are presented to clearly identify PCR analysis of newly synthesized pre-mRNA, as distinct from Pol II ChIP data.
(TIF)

S4 Fig Pol II ChIP analysis transcriptional activation from the dominant TSS of Nr4a1. Quantitative Pol II ChIP using input DNA equivalent to 200 genome copies. Pol II density estimates across the dominant transcriptional unit (2056–7623), averaged 17.3±5.2 copies (or 8.7±2.6%) during the plateau of activated expression (7–60 min). Single primers associated with TSS(c) at -11286 bp and the dominant TSS at 286 bp, provide uncorroborated evidence that promoter proximal density is elevated consistent with modest PPP after these promoters have been activated. Points not quantitated had inadequate reference curves (not determined; nd) or were below (<) the quantitation range.
(TIF)

S1 Table Microarray analysis. (XLS)

S2 Table Conserved IEG expression. (XLSX)

S3 Table ChIP primers. (XLS)

S4 Table Matrix ChIP primers. (XLS)

S5 Table TaqMan qPCR primers and data. (XLS)

## References

[1] Fuda NJ, Ardehali MB, Lis JT. Defining mechanisms that regulate RNA polymerase II transcription in vivo. Nature. 2009;461(7261):186–92. Epub 2009/09/11. doi: nature08449 [pii] 10.1038/nature08449 19741698 PMC2833331

[2] Venters BJ, Pugh BF. How eukaryotic genes are transcribed. Crit Rev Biochem Mol Biol. 2009;44(2–3):117–41. Epub 2009/06/12. 10.1080/10409230902858785 19514890 PMC2718758

[3] Keene JD. Minireview: global regulation and dynamics of ribonucleic Acid. Endocrinology. 2010;151(4):1391–7. Epub 2010/03/25. doi: 151/4/1391 [pii] 10.1210/en.2009-1250 20332203 PMC2850242

[4] Hampsey M, Singh BN, Ansari A, Laine JP, Krishnamurthy S. Control of eukaryotic gene expression: Gene loops and transcriptional memory. Adv Enzyme Regul. 2011;51(1):118–25. Epub 2010/11/03. doi: S0065-2571(10)00071-3 [pii] 10.1016/j.advenzreg.2010.10.001 . 21036187 PMC3305805

[5] Galbraith MD, Espinosa JM. Lessons on transcriptional control from the serum response network. Curr Opin Genet Dev. 2011;21(2):160–6. Epub 2011/02/15. doi: S0959-437X(11)00015-3 [pii] 10.1016/j.gde.2011.01.011 21316215 PMC3070842

[6] Nechaev S, Adelman K. Pol II waiting in the starting gates: Regulating the transition from transcription initiation into productive elongation. Biochim Biophys Acta. 2011;1809(1):34–45. Epub 2010/11/18. doi: S1874-9399(10)00139-2 [pii] 10.1016/j.bbagrm.2010.11.001 21081187 PMC3021596

[7] Munoz MJ, de la Mata M, Kornblihtt AR. The carboxy terminal domain of RNA polymerase II and alternative splicing. Trends Biochem Sci. 2010;35(9):497–504. Epub 2010/04/27. doi: S0968-0004(10)00069-1 [pii] 10.1016/j.tibs.2010.03.010 . 20418102

[8] Moore MJ, Proudfoot NJ. Pre-mRNA processing reaches back to transcription and ahead to translation. Cell. 2009;136(4):688–700. Epub 2009/02/26. doi: S0092-8674(09)00133-0 [pii] 10.1016/j.cell.2009.02.001 . 19239889

[9] Maniatis T, Reed R. An extensive network of coupling among gene expression machines. Nature. 2002;416(6880):499–506. Epub 2002/04/05. 10.1038/416499a 416499a [pii]. . 11932736

[10] Kim TH, Barrera LO, Zheng M, Qu C, Singer MA, Richmond TA, et al A high-resolution map of active promoters in the human genome. Nature. 2005;436(7052):876–80. Epub 2005/07/01. doi: nature03877 [pii] 10.1038/nature03877 15988478 PMC1895599

[11] Muse GW, Gilchrist DA, Nechaev S, Shah R, Parker JS, Grissom SF, et al RNA polymerase is poised for activation across the genome. Nat Genet. 2007;39(12):1507–11. Epub 2007/11/13. doi: ng.2007.21 [pii] 10.1038/ng.2007.21 17994021 PMC2365887

[12] Guenther MG, Levine SS, Boyer LA, Jaenisch R, Young RA. A chromatin landmark and transcription initiation at most promoters in human cells. Cell. 2007;130(1):77–88. Epub 2007/07/17. doi: S0092-8674(07)00681-2 [pii] 10.1016/j.cell.2007.05.042 . 17632057 PMC3200295

[13] Core LJ, Waterfall JJ, Lis JT. Nascent RNA sequencing reveals widespread pausing and divergent initiation at human promoters. Science. 2008;322(5909):1845–8. Epub 2008/12/06. doi: 1162228 [pii] 10.1126/science.1162228 19056941 PMC2833333

[14] Rahl PB, Lin CY, Seila AC, Flynn RA, McCuine S, Burge CB, et al c-Myc regulates transcriptional pause release. Cell. 2010;141(3):432–45. Epub 2010/05/04. doi: S0092-8674(10)00318-1 [pii] 10.1016/j.cell.2010.03.030 20434984 PMC2864022

[15] Nechaev S, Fargo DC, dos Santos G, Liu L, Gao Y, Adelman K. Global analysis of short RNAs reveals widespread promoter-proximal stalling and arrest of Pol II in Drosophila. Science. 2010;327(5963):335–8. Epub 2009/12/17. doi: science.1181421 [pii] 10.1126/science.1181421 . 20007866 PMC3435875

[16] Wada Y, Ohta Y, Xu M, Tsutsumi S, Minami T, Inoue K, et al A wave of nascent transcription on activated human genes. Proc Natl Acad Sci U S A. 2009;106(43):18357–61. Epub 2009/10/15. doi: 0902573106 [pii] 10.1073/pnas.0902573106 19826084 PMC2761237

[17] Gomes NP, Espinosa JM. Gene-specific repression of the p53 target gene PUMA via intragenic CTCF-Cohesin binding. Genes Dev. 2010;24(10):1022–34. Epub 2010/05/19. doi: 24/10/1022 [pii] 10.1101/gad.1881010 20478995 PMC2867207

[18] Kazerouninia A, Ngo B, Martinson HG. Poly(A) signal-dependent degradation of unprocessed nascent transcripts accompanies poly(A) signal-dependent transcriptional pausing in vitro. RNA. 2010;16(1):197–210. Epub 2009/11/21. doi: rna.1622010 [pii] 10.1261/rna.1622010 19926725 PMC2802029

[19] West S, Proudfoot NJ. Transcriptional termination enhances protein expression in human cells. Mol Cell. 2009;33(3):354–64. Epub 2009/02/17. doi: S1097-2765(09)00035-5 [pii] 10.1016/j.molcel.2009.01.008 19217409 PMC2706331

[20] Pandya-Jones A, Black DL. Co-transcriptional splicing of constitutive and alternative exons. RNA. 2009;15(10):1896–908. Epub 2009/08/07. doi: rna.1714509 [pii] 10.1261/rna.1714509 19656867 PMC2743041

[21] Zobeck KL, Buckley MS, Zipfel WR, Lis JT. Recruitment timing and dynamics of transcription factors at the Hsp70 loci in living cells. Mol Cell. 2010;40(6):965–75. Epub 2010/12/22. doi: S1097-2765(10)00888-9 [pii] 10.1016/j.molcel.2010.11.022 21172661 PMC3021954

[22] Seila AC, Core LJ, Lis JT, Sharp PA. Divergent transcription: a new feature of active promoters. Cell Cycle. 2009;8(16):2557–64. Epub 2009/07/15. doi: 9305 [pii]. . 19597342 10.4161/cc.8.16.9305

[23] Moran VA, Perera RJ, Khalil AM. Emerging functional and mechanistic paradigms of mammalian long non-coding RNAs. Nucleic Acids Res. 2012;40(14):6391–400. Epub 2012/04/12. 10.1093/nar/gks296 gks296 [pii]. 22492512 PMC3413108

[24] Muller R, Bravo R, Burckhardt J, Curran T. Induction of c-fos gene and protein by growth factors precedes activation of c-myc. Nature. 1984;312(5996):716–20. Epub 1984/12/20. . 6334806 10.1038/312716a0

[25] Greenberg ME, Ziff EB. Stimulation of 3T3 cells induces transcription of the c-fos proto-oncogene. Nature. 1984;311(5985):433–8. Epub 1984/10/04. . 6090941 10.1038/311433a0

[26] Iyer VR, Eisen MB, Ross DT, Schuler G, Moore T, Lee JC, et al The transcriptional program in the response of human fibroblasts to serum. Science. 1999;283(5398):83–7. Epub 1999/01/05 21:57. . 9872747 10.1126/science.283.5398.83

[27] Gasch AP. Comparative genomics of the environmental stress response in ascomycete fungi. Yeast. 2007;24(11):961–76. Epub 2007/07/03. 10.1002/yea.1512 . 17605132

[28] Westfall PJ, Patterson JC, Chen RE, Thorner J. Stress resistance and signal fidelity independent of nuclear MAPK function. Proc Natl Acad Sci U S A. 2008;105(34):12212–7. Epub 2008/08/23. doi: 0805797105 [pii] 10.1073/pnas.0805797105 18719124 PMC2518827

[29] Berry DB, Gasch AP. Stress-activated genomic expression changes serve a preparative role for impending stress in yeast. Mol Biol Cell. 2008;19(11):4580–7. Epub 2008/08/30. doi: E07-07-0680 [pii] 10.1091/mbc.E07-07-0680 18753408 PMC2575158

[30] Murry CE, Jennings RB, Reimer KA. Preconditioning with ischemia: a delay of lethal cell injury in ischemic myocardium. Circulation. 1986;74(5):1124–36. Epub 1986/11/01. . 3769170 10.1161/01.cir.74.5.1124

[31] Bolli R. Preconditioning: a paradigm shift in the biology of myocardial ischemia. Am J Physiol Heart Circ Physiol. 2007;292(1):H19–27. Epub 2006/09/12. doi: 00712.2006 [pii] 10.1152/ajpheart.00712.2006 . 16963615 PMC3242363

[32] Banerjee A, Locke-Winter C, Rogers KB, Mitchell MB, Brew EC, Cairns CB, et al Preconditioning against myocardial dysfunction after ischemia and reperfusion by an alpha 1-adrenergic mechanism. Circ Res. 1993;73(4):656–70. Epub 1993/10/01. . 8396503 10.1161/01.res.73.4.656

[33] Salvi S. Protecting the myocardium from ischemic injury: a critical role for alpha(1)-adrenoreceptors? Chest. 2001;119(4):1242–9. Epub 2001/04/11. . 11296192 10.1378/chest.119.4.1242

[34] Michelotti GA, Schwinn DA. Mechanistic insights into the role of alpha1adrenergic receptors in lower urinary tract symptoms. Curr Urol Rep. 2004;5(4):258–66. Epub 2004/07/21. . 15260925 10.1007/s11934-004-0048-0

[35] Cotecchia S. The alpha1-adrenergic receptors: diversity of signaling networks and regulation. J Recept Signal Transduct Res. 2010;30(6):410–9. Epub 2010/10/20. 10.3109/10799893.2010.518152 20954794 PMC3018134

[36] Suh BC, Hille B. PIP2 is a necessary cofactor for ion channel function: how and why? Annu Rev Biophys. 2008;37:175–95. Epub 2008/06/25. 10.1146/annurev.biophys.37.032807.125859 18573078 PMC2692585

[37] Lei B, Schwinn DA, Morris DP. Stimulation of alpha1a adrenergic receptors induces cellular proliferation or antiproliferative hypertrophy dependent solely on agonist concentration. PLoS One. 2013;8(8):e72430 Epub 2013/08/31. 10.1371/journal.pone.0072430 PONE-D-13-16240 [pii]. 23991110 PMC3749976

[38] Ahlquist RP. A study of the adrenotropic receptors. Am J Physiol. 1948;153(3):586–600. Epub 1948/06/01. . 18882199 10.1152/ajplegacy.1948.153.3.586

[39] Docherty JR. Subtypes of functional alpha1-adrenoceptor. Cell Mol Life Sci. 2010;67(3):405–17. Epub 2009/10/29. 10.1007/s00018-009-0174-4 . 19862476 PMC11115521

[40] O'Connell TD, Swigart PM, Rodrigo MC, Ishizaka S, Joho S, Turnbull L, et al Alpha1-adrenergic receptors prevent a maladaptive cardiac response to pressure overload. J Clin Invest. 2006;116(4):1005–15. Epub 2006/04/06. 10.1172/JCI22811 16585965 PMC1421341

[41] Huang Y, Wright CD, Merkwan CL, Baye NL, Liang Q, Simpson PC, et al An alpha1A-adrenergic-extracellular signal-regulated kinase survival signaling pathway in cardiac myocytes. Circulation. 2007;115(6):763–72. Epub 2007/02/07. doi: CIRCULATIONAHA.106.664862 [pii] 10.1161/CIRCULATIONAHA.106.664862 . 17283256

[42] Simpson PC. Role of proto-oncogenes in myocardial hypertrophy. Am J Cardiol. 1988;62(11):13G–9G. Epub 1988/10/05. . 2972186 10.1016/0002-9149(88)90026-4

[43] Autelitano DJ, Woodcock EA. Selective activation of alpha1A-adrenergic receptors in neonatal cardiac myocytes is sufficient to cause hypertrophy and differential regulation of alpha1-adrenergic receptor subtype mRNAs. J Mol Cell Cardiol. 1998;30(8):1515–23. Epub 1998/09/17. doi: S0022-2828(98)90717-9 [pii] 10.1006/jmcc.1998.0717 . 9737938

[44] Minneman KP, Lee D, Zhong H, Berts A, Abbott KL, Murphy TJ. Transcriptional responses to growth factor and G protein-coupled receptors in PC12 cells: comparison of alpha(1)-adrenergic receptor subtypes. J Neurochem. 2000;74(6):2392–400. Epub 2000/05/23. . 10820200 10.1046/j.1471-4159.2000.0742392.x

[45] Gonzalez-Cabrera PJ, Gaivin RJ, Yun J, Ross SA, Papay RS, McCune DF, et al Genetic profiling of alpha 1-adrenergic receptor subtypes by oligonucleotide microarrays: coupling to interleukin-6 secretion but differences in STAT3 phosphorylation and gp-130. Mol Pharmacol. 2003;63(5):1104–16. Epub 2003/04/16. . 12695539 10.1124/mol.63.5.1104

[46] Singh J, Padgett RA. Rates of in situ transcription and splicing in large human genes. Nat Struct Mol Biol. 2009;16(11):1128–33. Epub 2009/10/13. doi: nsmb.1666 [pii] 10.1038/nsmb.1666 19820712 PMC2783620

[47] Lei B, Morris DP, Smith MP, Svetkey LP, Newman MF, Rotter JI, et al Novel human alpha1a-adrenoceptor single nucleotide polymorphisms alter receptor pharmacology and biological function. Naunyn Schmiedebergs Arch Pharmacol. 2005;371(3):229–39. Epub 2005/05/19. 10.1007/s00210-005-1019-9 15900517 PMC2367253

[48] Morris DP, Michelotti GA, Schwinn DA. Evidence that phosphorylation of the RNA polymerase II carboxyl-terminal repeats is similar in yeast and humans. J Biol Chem. 2005;280(36):31368–77. Epub 2005/07/14. doi: M501546200 [pii] 10.1074/jbc.M501546200 16012166 PMC2277102

[49] Flanagin S, Nelson JD, Castner DG, Denisenko O, Bomsztyk K. Microplate-based chromatin immunoprecipitation method, Matrix ChIP: a platform to study signaling of complex genomic events. Nucleic Acids Res. 2008;36(3):e17 Epub 2008/01/22. 10.1093/nar/gkn001 gkn001 [pii]. 18203739 PMC2241906

[50] Saeed AE, Parmentier JH, Malik KU. Activation of alpha1A-adrenergic receptor promotes differentiation of rat-1 fibroblasts to a smooth muscle-like phenotype. BMC Cell Biol. 2004;5(1):47. Epub 2004/12/18. doi: 1471-2121-5-47 [pii] 10.1186/1471-2121-5-47 15603588 PMC548263

[51] Ruel M, Bianchi C, Khan TA, Xu S, Liddicoat JR, Voisine P, et al Gene expression profile after cardiopulmonary bypass and cardioplegic arrest. J Thorac Cardiovasc Surg. 2003;126(5):1521–30. Epub 2003/12/11. 10.1016/S0022S0022522303009693 [pii]. . 14666028

[52] Voisine P, Ruel M, Khan TA, Bianchi C, Xu SH, Kohane I, et al Differences in gene expression profiles of diabetic and nondiabetic patients undergoing cardiopulmonary bypass and cardioplegic arrest. Circulation. 2004;110(11 Suppl 1):II280–6. Epub 2004/09/15. 10.1161/01.CIR.0000138974.18839.02110/11_suppl_1/II-280 [pii]. . 15364876

[53] Ghorbel MT, Cherif M, Mokhtari A, Bruno VD, Caputo M, Angelini GD. Off-pump coronary artery bypass surgery is associated with fewer gene expression changes in the human myocardium in comparison with on-pump surgery. Physiol Genomics. 2010;42(1):67–75. Epub 2010/03/25. doi: physiolgenomics.00174.2009 [pii] 10.1152/physiolgenomics.00174.2009 20332183 PMC2888559

[54] Cullingford TE, Markou T, Fuller SJ, Giraldo A, Pikkarainen S, Zoumpoulidou G, et al Temporal regulation of expression of immediate early and second phase transcripts by endothelin-1 in cardiomyocytes. Genome Biol. 2008;9(2):R32. Epub 2008/02/16. doi: gb-2008-9-2-r32 [pii] 10.1186/gb-2008-9-2-r32 18275597 PMC2374717

[55] Mason PB, Struhl K. Distinction and relationship between elongation rate and processivity of RNA polymerase II in vivo. Mol Cell. 2005;17(6):831–40. Epub 2005/03/23. doi: S1097-2765(05)01116-0 [pii] 10.1016/j.molcel.2005.02.017 . 15780939

[56] Sandoval J, Rodriguez JL, Tur G, Serviddio G, Pereda J, Boukaba A, et al RNAPol-ChIP: a novel application of chromatin immunoprecipitation to the analysis of real-time gene transcription. Nucleic Acids Res. 2004;32(11):e88 Epub 2004/07/13. 10.1093/nar/gnh09132/11/e88 [pii]. 15247321 PMC443558

[57] Teytelman L, Ozaydin B, Zill O, Lefrancois P, Snyder M, Rine J, et al Impact of chromatin structures on DNA processing for genomic analyses. PLoS One. 2009;4(8):e6700 Epub 2009/08/21. 10.1371/journal.pone.0006700 19693276 PMC2725323

[58] Pinaud S, Mirkovitch J. Regulation of c-fos expression by RNA polymerase elongation competence. J Mol Biol. 1998;280(5):785–98. Epub 1998/07/22. doi: S0022-2836(98)91905-2 [pii] 10.1006/jmbi.1998.1905 . 9671550

[59] Fujita T, Ryser S, Piuz I, Schlegel W. Up-regulation of P-TEFb by the MEK1-extracellular signal-regulated kinase signaling pathway contributes to stimulated transcription elongation of immediate early genes in neuroendocrine cells. Mol Cell Biol. 2008;28(5):1630–43. Epub 2007/12/19. doi: MCB.01767-07 [pii] 10.1128/MCB.01767-07 18086894 PMC2258797

[60] Darnell JE Jr. Reflections on the history of pre-mRNA processing and highlights of current knowledge: a unified picture. RNA. 2013;19(4):443–60. Epub 2013/02/27. 10.1261/rna.038596.113 rna.038596.113 [pii]. 23440351 PMC3677254

[61] Fuchs G, Voichek Y, Benjamin S, Gilad S, Amit I, Oren M. 4sUDRB-seq: measuring genomewide transcriptional elongation rates and initiation frequencies within cells. Genome Biol. 2014;15(5):R69 Epub 2014/06/03. 10.1186/gb-2014-15-5-r69 gb-2014-15-5-r69 [pii]. 24887486 PMC4072947

[62] Kurakula K, Koenis DS, van Tiel CM, de Vries CJ. NR4A nuclear receptors are orphans but not lonesome. Biochim Biophys Acta. 2014;1843(11):2543–55. Epub 2014/07/01. doi: S0167-4889(14)00213-4 [pii] 10.1016/j.bbamcr.2014.06.010 . 24975497

[63] Pearen MA, Muscat GE. Minireview: Nuclear hormone receptor 4A signaling: implications for metabolic disease. Mol Endocrinol. 2010;24(10):1891–903. Epub 2010/04/16. doi: me.2010-0015 [pii] 10.1210/me.2010-0015 . 20392876 PMC5417389

[64] Weng YJ, Hsieh DJ, Kuo WW, Lai TY, Hsu HH, Tsai CH, et al E4BP4 is a cardiac survival factor and essential for embryonic heart development. Mol Cell Biochem. 2010;340(1–2):187–94. Epub 2010/02/27. 10.1007/s11010-010-0417-6 . 20186462

[65] Glover-Cutter K, Kim S, Espinosa J, Bentley DL. RNA polymerase II pauses and associates with pre-mRNA processing factors at both ends of genes. Nat Struct Mol Biol. 2008;15(1):71–8. Epub 2007/12/25. doi: nsmb1352 [pii] 10.1038/nsmb1352 18157150 PMC2836588

[66] Grosso AR, de Almeida SF, Braga J, Carmo-Fonseca M. Dynamic transitions in RNA polymerase II density profiles during transcription termination. Genome Res. 2012;22(8):1447–56. Epub 2012/06/12. 10.1101/gr.138057.112 gr.138057.112 [pii]. 22684278 PMC3409258

[67] Caunt CJ, Keyse SM. Dual-specificity MAP kinase phosphatases (MKPs): shaping the outcome of MAP kinase signalling. FEBS J. 2013;280(2):489–504. Epub 2012/07/21. 10.1111/j.1742-4658.2012.08716.x 22812510 PMC3594966

[68] Kadara H, Fujimoto J, Men T, Ye X, Lotan D, Lee JS, et al A Gprc5a tumor suppressor loss of expression signature is conserved, prevalent, and associated with survival in human lung adenocarcinomas. Neoplasia. 2010;12(6):499–505. Epub 2010/06/22. 20563252 10.1593/neo.10390 PMC2887090

[69] Spriggs KA, Bushell M, Willis AE. Translational regulation of gene expression during conditions of cell stress. Mol Cell. 2010;40(2):228–37. Epub 2010/10/23. doi: S1097-2765(10)00756-2 [pii] 10.1016/j.molcel.2010.09.028 . 20965418

[70] Uesono Y, Toh EA. Transient inhibition of translation initiation by osmotic stress. J Biol Chem. 2002;277(16):13848–55. Epub 2002/01/18. 10.1074/jbc.M108848200 M108848200 [pii]. . 11796711

[71] Krumm A, Hickey LB, Groudine M. Promoter-proximal pausing of RNA polymerase II defines a general rate-limiting step after transcription initiation. Genes Dev. 1995;9(5):559–72. Epub 1995/03/01. . 7698646 10.1101/gad.9.5.559

[72] Eick D, Kohlhuber F, Wolf DA, Strobl LJ. Activation of pausing RNA polymerases by nuclear run-on experiments. Anal Biochem. 1994;218(2):347–51. Epub 1994/05/01. doi: S0003269784711900 [pii]. . 8074291 10.1006/abio.1994.1190

[73] Rougvie AE, Lis JT. Postinitiation transcriptional control in Drosophila melanogaster. Mol Cell Biol. 1990;10(11):6041–5. Epub 1990/11/01. 2172790 10.1128/mcb.10.11.6041-6045.1990 PMC361402

[74] Jencks WP. On the attribution and additivity of binding energies. Proc Natl Acad Sci U S A. 1981;78(7):4046–50. Epub 1981/07/01. 16593049 10.1073/pnas.78.7.4046 PMC319722

[75] Cho EJ, Kobor MS, Kim M, Greenblatt J, Buratowski S. Opposing effects of Ctk1 kinase and Fcp1 phosphatase at Ser 2 of the RNA polymerase II C-terminal domain. Genes Dev. 2001;15(24):3319–29. Epub 2001/12/26. 10.1101/gad.935901 11751637 PMC312848

[76] Jones JC, Phatnani HP, Haystead TA, MacDonald JA, Alam SM, Greenleaf AL. C-terminal repeat domain kinase I phosphorylates Ser2 and Ser5 of RNA polymerase II C-terminal domain repeats. J Biol Chem. 2004;279(24):24957–64. Epub 2004/03/30. 10.1074/jbc.M402218200 M402218200 [pii]. 15047695 PMC2680323

[77] Komarnitsky P, Cho EJ, Buratowski S. Different phosphorylated forms of RNA polymerase II and associated mRNA processing factors during transcription. Genes Dev. 2000;14(19):2452–60. Epub 2000/10/06. 11018013 10.1101/gad.824700 PMC316976

[78] Kim M, Suh H, Cho EJ, Buratowski S. Phosphorylation of the yeast Rpb1 C-terminal domain at serines 2, 5, and 7. J Biol Chem. 2009;284(39):26421–6. Epub 2009/08/15. doi: M109.028993 [pii] 10.1074/jbc.M109.028993 19679665 PMC2785330

[79] Tietjen JR, Zhang DW, Rodriguez-Molina JB, White BE, Akhtar MS, Heidemann M, et al Chemical-genomic dissection of the CTD code. Nat Struct Mol Biol. 2010;17(9):1154–61. Epub 2010/08/31. doi: nsmb.1900 [pii] 10.1038/nsmb.1900 . 20802488 PMC4035229

[80] Mayer A, Lidschreiber M, Siebert M, Leike K, Soding J, Cramer P. Uniform transitions of the general RNA polymerase II transcription complex. Nat Struct Mol Biol. 2010;17(10):1272–8. Epub 2010/09/08. doi: nsmb.1903 [pii] 10.1038/nsmb.1903 . 20818391

[81] Kim H, Erickson B, Luo W, Seward D, Graber JH, Pollock DD, et al Gene-specific RNA polymerase II phosphorylation and the CTD code. Nat Struct Mol Biol. 2010;17(10):1279–86. Epub 2010/09/14. doi: nsmb.1913 [pii] 10.1038/nsmb.1913 20835241 PMC3048030

[82] Chapman RD, Heidemann M, Albert TK, Mailhammer R, Flatley A, Meisterernst M, et al Transcribing RNA polymerase II is phosphorylated at CTD residue serine-7. Science. 2007;318(5857):1780–2. Epub 2007/12/15. doi: 318/5857/1780 [pii] 10.1126/science.1145977 . 18079404

[83] Cheng C, Sharp PA. RNA polymerase II accumulation in the promoter-proximal region of the dihydrofolate reductase and gamma-actin genes. Mol Cell Biol. 2003;23(6):1961–7. Epub 2003/03/04. 12612070 10.1128/MCB.23.6.1961-1967.2003 PMC149466

[84] Fujita T, Ryser S, Tortola S, Piuz I, Schlegel W. Gene-specific recruitment of positive and negative elongation factors during stimulated transcription of the MKP-1 gene in neuroendocrine cells. Nucleic Acids Res. 2007;35(3):1007–17. Epub 2007/01/30. doi: gkl1138 [pii] 10.1093/nar/gkl1138 17259211 PMC1807974

[85] Gomes NP, Bjerke G, Llorente B, Szostek SA, Emerson BM, Espinosa JM. Gene-specific requirement for P-TEFb activity and RNA polymerase II phosphorylation within the p53 transcriptional program. Genes Dev. 2006;20(5):601–12. Epub 2006/03/03. doi: 20/5/601 [pii] 10.1101/gad.1398206 16510875 PMC1410802

[86] Donner AJ, Szostek S, Hoover JM, Espinosa JM. CDK8 is a stimulus-specific positive coregulator of p53 target genes. Mol Cell. 2007;27(1):121–33. Epub 2007/07/07. doi: S1097-2765(07)00327-9 [pii] 10.1016/j.molcel.2007.05.026 17612495 PMC2936241

[87] Donner AJ, Ebmeier CC, Taatjes DJ, Espinosa JM. CDK8 is a positive regulator of transcriptional elongation within the serum response network. Nat Struct Mol Biol. 2010;17(2):194–201. Epub 2010/01/26. doi: nsmb.1752 [pii] 10.1038/nsmb.1752 20098423 PMC2920286

[88] Arner E, Daub CO, Vitting-Seerup K, Andersson R, Lilje B, Drablos F, et al Gene regulation. Transcribed enhancers lead waves of coordinated transcription in transitioning mammalian cells. Science. 2015;347(6225):1010–4. Epub 2015/02/14. 10.1126/science.1259418 . 25678556 PMC4681433

[89] Fujita T, Schlegel W. Promoter-proximal pausing of RNA polymerase II: an opportunity to regulate gene transcription. J Recept Signal Transduct Res. 2010;30(1):31–42. Epub 2010/02/23. 10.3109/10799890903517921 . 20170405

[90] Connelly S, Manley JL. A functional mRNA polyadenylation signal is required for transcription termination by RNA polymerase II. Genes Dev. 1988;2(4):440–52. Epub 1988/04/01. . 2836265 10.1101/gad.2.4.440

[91] Edwalds-Gilbert G, Prescott J, Falck-Pedersen E. 3' RNA processing efficiency plays a primary role in generating termination-competent RNA polymerase II elongation complexes. Mol Cell Biol. 1993;13(6):3472–80. Epub 1993/06/01. 7684499 10.1128/mcb.13.6.3472-3480.1993 PMC359816

[92] Peterson ML, Bertolino S, Davis F. An RNA polymerase pause site is associated with the immunoglobulin mus poly(A) site. Mol Cell Biol. 2002;22(15):5606–15. Epub 2002/07/09. 12101252 10.1128/MCB.22.15.5606-5615.2002 PMC133935

[93] Orozco IJ, Kim SJ, Martinson HG. The poly(A) signal, without the assistance of any downstream element, directs RNA polymerase II to pause in vivo and then to release stochastically from the template. J Biol Chem. 2002;277(45):42899–911. Epub 2002/08/28. 10.1074/jbc.M207415200 M207415200 [pii]. . 12196547

[94] Nag A, Narsinh K, Kazerouninia A, Martinson HG. The conserved AAUAAA hexamer of the poly(A) signal can act alone to trigger a stable decrease in RNA polymerase II transcription velocity. RNA. 2006;12(8):1534–44. Epub 2006/06/16. doi: rna.103206 [pii] 10.1261/rna.103206 16775304 PMC1524889

[95] Nag A, Narsinh K, Martinson HG. The poly(A)-dependent transcriptional pause is mediated by CPSF acting on the body of the polymerase. Nat Struct Mol Biol. 2007;14(7):662–9. Epub 2007/06/19. doi: nsmb1253 [pii] 10.1038/nsmb1253 . 17572685

[96] Chao LC, Jamil A, Kim SJ, Huang L, Martinson HG. Assembly of the cleavage and polyadenylation apparatus requires about 10 seconds in vivo and is faster for strong than for weak poly(A) sites. Mol Cell Biol. 1999;19(8):5588–600. Epub 1999/07/20. 10409748 10.1128/mcb.19.8.5588 PMC84411

[97] Carswell S, Alwine JC. Efficiency of utilization of the simian virus 40 late polyadenylation site: effects of upstream sequences. Mol Cell Biol. 1989;9(10):4248–58. Epub 1989/10/01. 2573828 10.1128/mcb.9.10.4248-4258.1989 PMC362504

[98] Hughes TA. Regulation of gene expression by alternative untranslated regions. Trends Genet. 2006;22(3):119–22. Epub 2006/01/25. doi: S0168-9525(06)00002-3 [pii] 10.1016/j.tig.2006.01.001 . 16430990

[99] Darzacq X, Shav-Tal Y, de Turris V, Brody Y, Shenoy SM, Phair RD, et al In vivo dynamics of RNA polymerase II transcription. Nat Struct Mol Biol. 2007;14(9):796–806. Epub 2007/08/07. doi: nsmb1280 [pii] 10.1038/nsmb1280 . 17676063 PMC4942130

[100] Ardehali MB, Lis JT. Tracking rates of transcription and splicing in vivo. Nat Struct Mol Biol. 2009;16(11):1123–4. Epub 2009/11/06. doi: nsmb1109-1123 [pii] 10.1038/nsmb1109-1123 . 19888309

[101] Tennyson CN, Klamut HJ, Worton RG. The human dystrophin gene requires 16 hours to be transcribed and is cotranscriptionally spliced. Nat Genet. 1995;9(2):184–90. Epub 1995/02/01. 10.1038/ng0295-184 . 7719347

[102] Danko CG, Hah N, Luo X, Martins AL, Core L, Lis JT, et al Signaling pathways differentially affect RNA polymerase II initiation, pausing, and elongation rate in cells. Mol Cell. 2013;50(2):212–22. Epub 2013/03/26. 10.1016/j.molcel.2013.02.015 S1097-2765(13)00171-8 [pii]. 23523369 PMC3640649

[103] Kwak H, Fuda NJ, Core LJ, Lis JT. Precise maps of RNA polymerase reveal how promoters direct initiation and pausing. Science. 2013;339(6122):950–3. Epub 2013/02/23. 10.1126/science.1229386 339/6122/950 [pii]. 23430654 PMC3974810

[104] Niwa M, Rose SD, Berget SM. In vitro polyadenylation is stimulated by the presence of an upstream intron. Genes Dev. 1990;4(9):1552–9. Epub 1990/09/01. . 1701407 10.1101/gad.4.9.1552

[105] Cooke C, Hans H, Alwine JC. Utilization of splicing elements and polyadenylation signal elements in the coupling of polyadenylation and last-intron removal. Mol Cell Biol. 1999;19(7):4971–9. Epub 1999/06/22. 10373547 10.1128/mcb.19.7.4971 PMC84315

[106] Kyburz A, Friedlein A, Langen H, Keller W. Direct interactions between subunits of CPSF and the U2 snRNP contribute to the coupling of pre-mRNA 3' end processing and splicing. Mol Cell. 2006;23(2):195–205. Epub 2006/07/22. doi: S1097-2765(06)00379-0 [pii] 10.1016/j.molcel.2006.05.037 . 16857586

[107] Millevoi S, Loulergue C, Dettwiler S, Karaa SZ, Keller W, Antoniou M, et al An interaction between U2AF 65 and CF I(m) links the splicing and 3' end processing machineries. EMBO J. 2006;25(20):4854–64. Epub 2006/10/07. doi: 7601331 [pii] 10.1038/sj.emboj.7601331 17024186 PMC1618107

[108] Rigo F, Martinson HG. Functional coupling of last-intron splicing and 3'-end processing to transcription in vitro: the poly(A) signal couples to splicing before committing to cleavage. Mol Cell Biol. 2008;28(2):849–62. Epub 2007/10/31. doi: MCB.01410-07 [pii] 10.1128/MCB.01410-07 17967872 PMC2223410

[109] Rigo F, Martinson HG. Polyadenylation releases mRNA from RNA polymerase II in a process that is licensed by splicing. RNA. 2009;15(5):823–36. Epub 2009/03/24. doi: rna.1409209 [pii] 10.1261/rna.1409209 19304926 PMC2673064

[110] Severin J, Lizio M, Harshbarger J, Kawaji H, Daub CO, Hayashizaki Y, et al Interactive visualization and analysis of large-scale sequencing datasets using ZENBU. Nature biotechnology. 2014;32(3):217–9. Epub 2014/04/15. 10.1038/nbt.2840 . 24727769

[111] Woodcock EA, Du XJ, Reichelt ME, Graham RM. Cardiac alpha 1-adrenergic drive in pathological remodelling. Cardiovasc Res. 2008;77(3):452–62. Epub 2007/11/23. doi: cvm078 [pii] 10.1093/cvr/cvm078 . 18032391

[112] O'Connell TD, Ishizaka S, Nakamura A, Swigart PM, Rodrigo MC, Simpson GL, et al The alpha(1A/C)- and alpha(1B)-adrenergic receptors are required for physiological cardiac hypertrophy in the double-knockout mouse. J Clin Invest. 2003;111(11):1783–91. Epub 2003/06/05. 10.1172/JCI16100 12782680 PMC156101

