# Correction: Temporal Dissection of Rate Limiting Transcriptional Events Using Pol II ChIP and RNA Analysis of Adrenergic Stress Gene Activation

**DOI:** 10.1371/journal.pone.0140710

**Published:** 2015-10-09

**Authors:** The PLOS ONE Staff

Multiple panels are missing from S1 and S2 Figs due to errors that occurred during the typesetting process. The publisher apologizes for these errors. Please view the correct S1 and S2 Figs below.

## Supporting Information

S1 Fig Analysis of Pol II density across the transcriptional units following gene activation. ChIP density estimates of Pol II density during the indicated period of maximal, activated expression following α_1a_AR stimulation. Primer locations associated with 5’ end of upstream primer relative to the transcription start site (i.e. TSS bp is 1). (**A**) Pol II density on Fos, expressed as the number of precipitated DNA copies from a maximum of 200 copies, was constant across proximal and distal regions indicating a complete abrogation of proximal pausing. (**B**) Elevated promoter proximal Pol II density on Nr4a3 (500 copies max.) shows incomplete abrogation of pausing, while a distal decrease in density was reported by regression analysis as a negative slope (-0.28 copies/kbp) that was statistically significant (p = 0.022). Lower density in distal regions is a consequence of partial transcription termination at an internal polyadenylation site at 14,004 bp. (**C**) Pol II density across the dominant transcriptional unit of Nr4a1 (200 copies max) displays no systematic variation; however, the TSS appears to be associated with modest promoter proximal pausing. (**D**) For Dusp5 (500 copies max), elevated promoter proximal density even after activation suggests only partial abrogation of pausing. (**E**) Density across Gpcr5a shows no systematic variation in Pol II density following abrogation of an internal transcriptional pause. Because the values from each primer are not independent (due to use of a common quantitation curve), error bars show standard deviation. Linear regression analysis was done using the average value observed for each primer pair within the time period indicated.
(TIF)

S2 Fig Analysis of Pol II density at times following α_1a_AR stimulation. Combining quantifiable Pol II ChIP density estimates for many primers at each time point provides a measure of aggregate Pol II density on the gene. (A) Across the Fos gene, Pol II density, expressed as the number of precipitated DNA copies from a maximum of 200 possible copies, was constant between 7 and 60 minutes but showed decreased transcription at 120 minutes (n = 5). (B) For Nr4a3 (500 copies max), polymerase density (beyond promoter effects) was consistently higher upstream of the internal polyadenylation site at 14,004 bp; however, no significant temporal difference was observed in either proximal (n = 6–7) or distal (n = 8) regions [nonquantifiable time points were essentially zero]. (C) For Nr4a1 (200 copies max.), non-proximal Pol II density across the dominant transcriptional unit was constant from 7 to 120 minutes (n = 7). (D) Non-proximal density on Dusp5 (500 copies max.) was constant from 7 to 60 minutes but decreased by 120 minutes (n = 12). (E) For Nfil3 no significant difference in non-proximal Pol II densities (expressed as % ChIP efficiency) was found between 10 and 120 minutes (n = 15), nevertheless regression analysis showed an increase in slope (0.025±0.11% ChIP eff./min) that was statistically significant (p = 0.032) during the first hour following α1aAR stimulation. Individual density estimates for each point are independent as they were produced using different primers and quantititation curves. Consequently, linear regression analysis was performed using individual data values and the error bars show SEM. One-way ANOVA analysis with the Turkey post-test was used to demonstrate statistical difference and identify time points with non-maximal density (open circles). To avoid trivial statistical difference, early time points with basal polymerase density were not included in the statistical analysis.
(TIF)
