# Supplementary figures and images for: Temporal Dissection of Rate Limiting Transcriptional Events Using Pol II ChIP and RNA Analysis of Adrenergic Stress Gene Activation
Source: PLoS One. 2015 Aug 5;10(8):e0134442. doi: 10.1371/journal.pone.0134442 (PMC4526373; doi:10.1371/journal.pone.0134442)

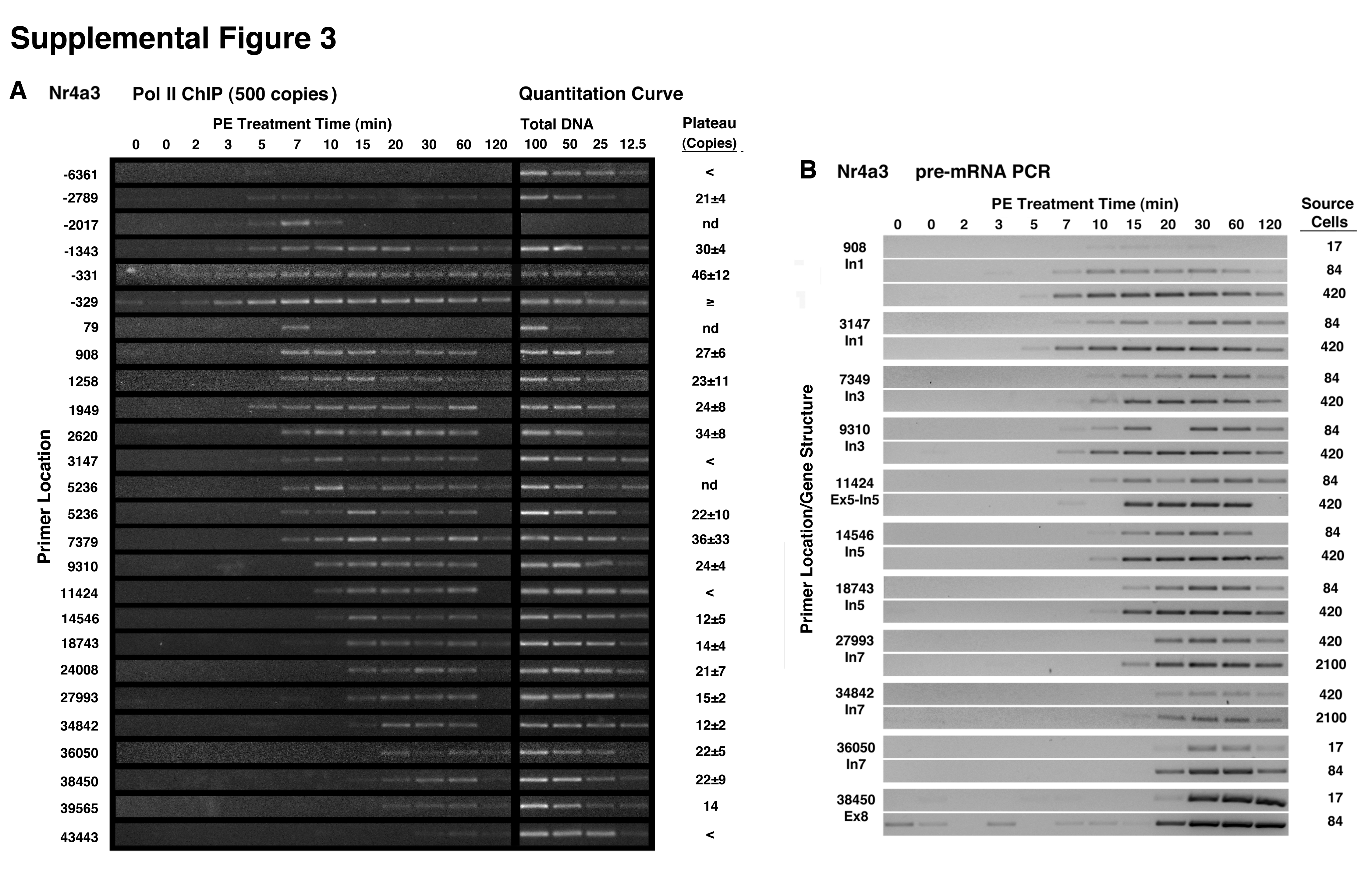

Supplement: S3 Fig — (A) Quantitative Pol II ChIP using input DNA equivalent to 200 genome copies. Pol II density is within the dynamic range and quantitative estimates of ChIPed DNA copies (right) during the plateau of activated expression suggests density is modestly lower in more distal regions (14546–39565). Points not quantitated had inadequate reference curves (not determined; nd) or were above (≥) or below (<) the quantitation range. (B) Qualitative PCR analysis of Nr4a3 pre-mRNA synthesis using ChIP primers. Analysis of randomly-primed total RNA reveals initial pre-mRNA production corresponds very closely to arrival of the dominant wave of Pol II apparent in the prior panel. The analysis also shows the near absence of basal Nr4a3 transcription. The number of source cells used to produce cDNA for each profile is shown at the far left, with 2,100 cells corresponds to 12.5 ng of total RNA. Negative images are presented to clearly identify PCR analysis of newly synthesized pre-mRNA, as distinct from Pol II ChIP data. (TIF) [file pone.0134442.s003.tif]

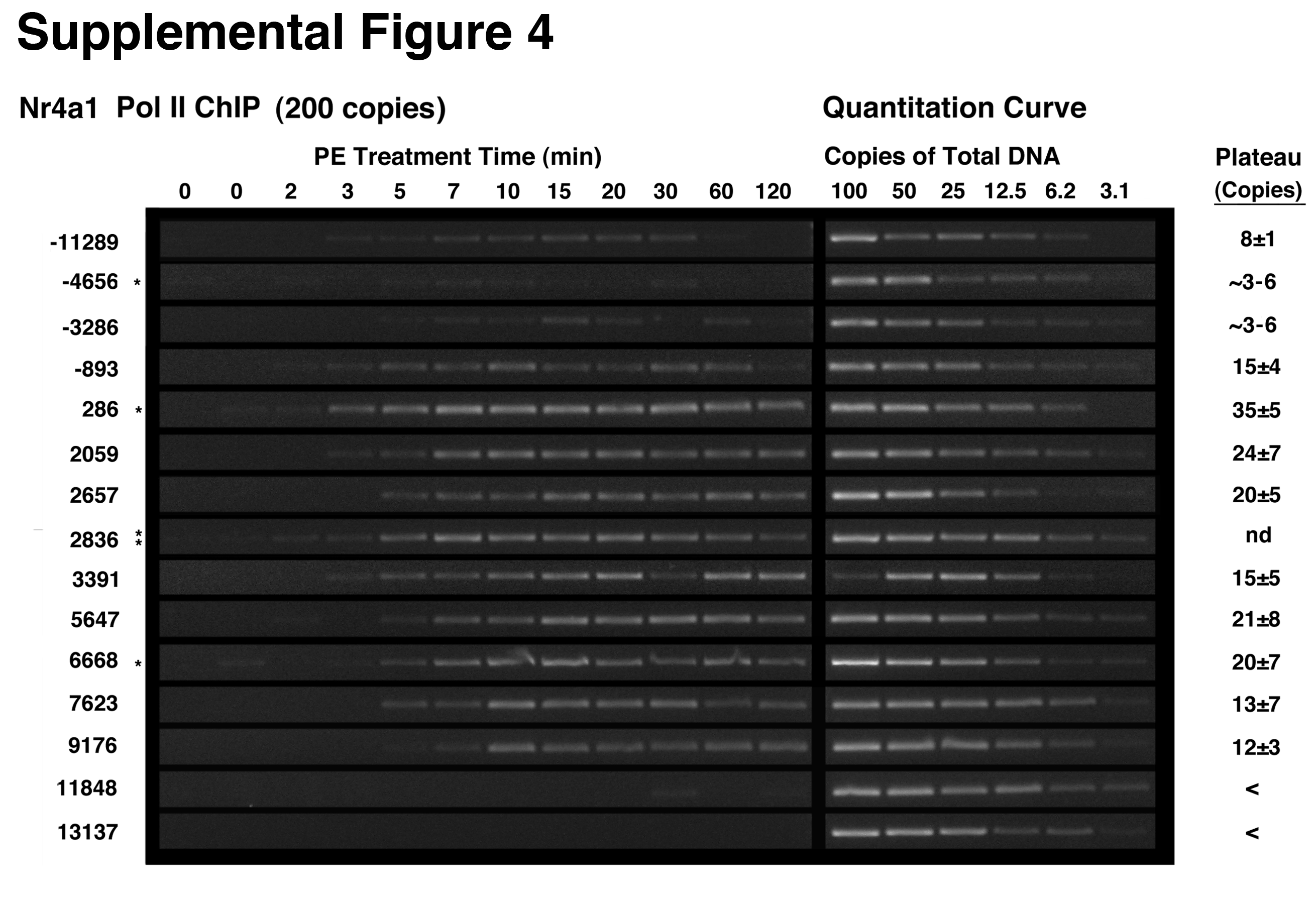

Supplement: S4 Fig — Quantitative Pol II ChIP using input DNA equivalent to 200 genome copies. Pol II density estimates across the dominant transcriptional unit (2056–7623), averaged 17.3±5.2 copies (or 8.7±2.6%) during the plateau of activated expression (7–60 min). Single primers associated with TSS(c) at -11286 bp and the dominant TSS at 286 bp, provide uncorroborated evidence that promoter proximal density is elevated consistent with modest PPP after these promoters have been activated. Points not quantitated had inadequate reference curves (not determined; nd) or were below (<) the quantitation range. (TIF) [file pone.0134442.s004.tif]
